# XY0206 targets FLT3-dependent resistance states in acute myeloid leukemia

**DOI:** 10.1172/JCI204701

**Published:** 2026-09-01

**Authors:** Long Shen, Yang Yang, Chenghua Xu, Bo Jiang, Xiaoxiao Duan, Xianfeng Shao, Simeng Li, Siyi Liu, Chao Huang, Lin Song, Mingyuan Sun, Jinting Fan, Ning Wang, Dong Zhang, Youyang Fang, Lichun Kang, Yajun Jiang, Mingming Niu, Junyuan Qi, Tao Cheng, Hong Wang

**Affiliations:** 1 State Key Laboratory of Experimental Hematology, National Clinical Research Center for Blood Diseases, Haihe Laboratory of Cell Ecosystem, Institute of Hematology & Blood Diseases Hospital, Chinese Academy of Medical Sciences & Peking Union Medical College, Tianjin, China.; 2 Tianjin Institutes of Health Science, Tianjin, China.; 3 Haihe Laboratory of Cell Ecosystem, Tianjin Medical University, Tianjin, China.; 4 State Key Laboratory of Pharmaceutical Biotechnology, School of Life Sciences, Chemistry and Biomedicine Innovation Center (ChemBIC), Nanjing University, Nanjing, China.; 5 Department of Clinical Laboratory, Tianjin First Central Hospital, School of Medicine, Nankai University, Tianjin, China.

**Keywords:** Cell biology, Clinical Research, Hematology, Clinical trials, Drug therapy, Leukemias

## Abstract

Activating mutations in FMS-like tyrosine kinase 3 (FLT3) drive aggressive acute myeloid leukemia (AML) and confer poor prognosis. Although FLT3 inhibitors have improved AML outcomes, their efficacy is frequently limited by microenvironment-mediated signaling and treatment-emergent resistance. XY0206 is a structurally optimized derivative of sunitinib, an inhibitor approved for multiple solid tumors. Biochemical, multi-omics, and functional analyses showed that XY0206 directly engages FLT3 and suppresses downstream STAT5, AKT, and ERK signaling, resulting in apoptosis of AML cells with *FLT3*–internal tandem duplications (*FLT3*-ITD). Across models of FLT3-dependent resistance, XY0206 retained antileukemic activity, including in *FLT3*-ITD cells harboring the F691L gatekeeper mutation, a recurrent alteration conferring resistance to approved FLT3 inhibitors. In primary AML blasts and xenograft models, XY0206 exhibited enhanced antileukemic activity with favorable tolerability relative to gilteritinib. In a phase I/II trial of XY0206 monotherapy in patients with relapsed or refractory (R/R) AML (ClinicalTrials.gov identifier NCT04471064), XY0206 achieved a composite complete remission rate (CRc) of 45.7% overall, with a notable 60.0% CRc rate among patients with *FLT3*-ITD mutations. Three of 8 patients with prior FLT3 inhibitor–exposed R/R AML also achieved CRc. Together, these findings support further clinical evaluation of XY0206 as an FLT3-directed therapeutic in AML, particularly in disease settings with reduced sensitivity to existing FLT3 inhibitors.

## Abstract 2

XY0206 shows promise as a targeted treatment for acute leukemia, including cases resistant to existing FLT3 inhibitors.

## Introduction

Acute myeloid leukemia (AML) is a highly aggressive hematologic malignancy and represents the most common type of acute leukemia in adults (1, 2). Approximately 30% of newly diagnosed AML cases harbor mutations in the *FLT3* gene, most commonly internal tandem duplications (*FLT3*-ITD) or point mutations within the tyrosine kinase domain (*FLT3*-TKD) (3). These mutations lead to ligand-independent FLT3 activation and stimulate downstream STAT5, PI3K/AKT, and RAS/MAPK/ERK signaling pathways. Sustained activation of these signaling programs promotes leukemic cell proliferation and survival and is associated with adverse clinical outcomes and therapeutic resistance (4, 5).

Several FLT3 inhibitors (FLT3is), including midostaurin, quizartinib, and gilteritinib, have improved treatment outcomes in AML with *FLT3* mutations (6–8). However, durable disease control remains limited, and resistance represents a substantial therapeutic challenge for all approved FLT3is (9). Despite continued efforts to develop next-generation FLT3is, many candidates remain in early development or have failed to progress clinically, highlighting the difficulty of generating FLT3-directed agents that retain activity in resistant disease states.

The underlying cause of drug resistance is the persistence of residual leukemic cells after initial treatment. Resistance can arise through microenvironment-derived survival signals, treatment-emergent on-target *FLT3* mutations, or off-target genetic alterations that reduce FLT3 dependence (10–13). Among the on-target mechanisms, the *FLT3*-F691L gatekeeper mutation is particularly problematic because it confers cross-resistance to all currently approved FLT3is (14, 15). Although emerging agents such as FF-10101 demonstrate preclinical activity against this mutation, few have progressed beyond early clinical evaluation, underscoring the continued need for therapeutic strategies capable of addressing both microenvironment-associated and mutation-driven resistance mechanisms (16–22).

To address these limitations, we investigated XY0206, a structurally optimized derivative of sunitinib. Sunitinib is a clinically approved multi-kinase inhibitor with potent activity against several RTKs, including FLT3 (23), but its broad kinase profile contributes to off-target toxicities that limit its applicability in hematologic malignancies (24, 25). XY0206 was designed to preserve FLT3-directed activity while improving the pharmacologic profile of the sunitinib scaffold. In this study, we comprehensively characterized the molecular mechanism and therapeutic potential of XY0206 in *FLT3*-mutated AML.

Using biochemical kinase profiling, integrated proteomic and phosphoproteomic profiling, cellular and mouse models, and early-phase clinical data, we show that XY0206 preferentially suppresses FLT3-centered signaling and retains antileukemic activity in multiple FLT3-dependent resistance contexts, including growth factor–associated resistance, acquired resistance to FLT3 inhibition, and *FLT3*-F691L–driven resistance. In a phase I/II clinical trial of XY0206 monotherapy for patients with relapsed or refractory (R/R) AML, XY0206 demonstrated tolerability and preliminary antileukemic activity, particularly in patients with *FLT3*-ITD mutations. Clinical benefit was also observed in a subset of patients whose AML had relapsed or become refractory after prior FLT3i-based therapies, providing translational support for the activity of XY0206 in FLT3i-exposed disease. Together, these results position XY0206 as a structurally and mechanistically distinct FLT3-directed therapeutic candidate and provide a rationale for its continued clinical evaluation in *FLT3*-mutated AML.

## Results

### Biochemical and multi-omics profiling define an FLT3-centered inhibitory profile of XY0206.

XY0206, originally reported as compound 10d, was developed through structure-guided optimization of the sunitinib scaffold to improve FLT3-directed potency and tolerability (Figure 1A). This optimization focused on the solvent-interface region of sunitinib, where modification of the *N*-diethylaminoethyl carboxamide tail was intended to improve the potency and tolerability of the sunitinib scaffold while preserving activity against FLT3-dependent AML. Prior work demonstrated potent FLT3/FLT3-ITD inhibition, preferential activity in *FLT3*-ITD AML models, favorable oral pharmacokinetics, and improved tolerability compared with sunitinib (26).

To further define the biochemical activity spectrum of XY0206, we performed single-concentration kinase profiling at 100 nM against a panel of 217 kinases. This assay was used to broadly assess kinase coverage at a concentration substantially above the reported FLT3/FLT3-ITD IC_50_ values, allowing identification of additional potential kinase targets. Under this condition, XY0206 strongly inhibited FLT3, along with other RTKs, including RET and PDGFRβ, with near-complete inhibition observed (Figure 1B and Supplemental Table 1; supplemental material available online with this article; https://doi.org/10.1172/JCI204701DS1), whereas the majority of kinases in the panel showed lower levels of inhibition. These data support an FLT3-centered kinase inhibition profile for XY0206, with additional activity against selected kinases under this screening condition.

We then integrated previously reported and newly generated enzymatic IC_50_ data for representative RTKs, selected based on the kinase profiling results and the known sunitinib target spectrum (26). XY0206 most potently inhibited FLT3-ITD (IC_50_ = 0.70 nM), followed by FLT3 and RET (IC_50_ = 5.27 nM and 6.92 nM, respectively). Activity against PDGFRβ was lower (IC_50_ = 26.00 nM), whereas inhibition of PDGFRα, KDR, KIT, and ALK was substantially weaker (IC_50_ = 158–675 nM) (Table 1). Compared with FLT3-ITD, XY0206 was ~37-fold less potent against PDGFRβ and >200-fold less potent against PDGFRα, KDR, KIT, and ALK, highlighting its preferential biochemical inhibition of FLT3-ITD while retaining modest activity against RET and PDGFRβ. Because RET has been implicated in supporting FLT3-ITD stability and PDGFRβ can activate downstream AKT and ERK signaling pathways that overlap with FLT3 signaling (27, 28), these data support preferential targeting of an FLT3-centered RTK signaling network.

To define the cellular consequences of XY0206-mediated FLT3-centered kinase inhibition, we performed integrated proteomic and phosphoproteomic analyses in *FLT3*-ITD AML cells treated with matched concentrations of XY0206 or sunitinib (Figure 1C). Proteomic profiling identified distinct drug-regulated protein expression programs following XY0206 or sunitinib treatment (Figure 1D and Supplemental Table 2). Pathway-level analysis of the proteomic data indicated suppression of AML- and proliferation-associated signaling programs, including cell-cycle, FLT3, MAPK, and PI3K/AKT/mTOR pathways (Figure 1E), consistent with attenuation of FLT3-dependent proliferative and survival signaling. Under matched treatment conditions, sunitinib showed comparatively stronger modulation of broader RTK- and NOTCH-associated pathways, consistent with its multi-kinase activity profile.

Global phosphoproteomic profiling was then used to define kinase-substrate-level signaling changes. XY0206 treatment was associated with reduced phosphorylation of multiple FLT3 downstream intermediates, including MAP2K2, PTPN11, SOS1, and STAT5A (Supplemental Figure 1, A and B, and Supplemental Table 3). These site-specific phosphorylation changes were further integrated at the pathway level, revealing coordinated modulation of apoptosis, cell death, cell differentiation, cell division, and cell proliferation programs, together with canonical FLT3 downstream signaling (Figure 1F), indicating coordinated modulation of antileukemic biological programs. Under matched treatment conditions, sunitinib had less pronounced effects on these FLT3-associated phosphoproteomic programs.

Kinase activity was inferred from phosphosite dynamics using the KSEA and RoKAI apps. To more directly interrogate FLT3/RTK-associated signaling, we additionally performed phosphotyrosine-enriched phosphoproteomic profiling, which increased the number of detected tyrosine-phosphorylated sites from 1,783 in the global phosphoproteomic dataset to 7,515 (Supplemental Figure 1C and Supplemental Table 4). The resulting phosphotyrosine-enriched kinase analysis showed that XY0206-modulated kinases were primarily concentrated in FLT3/RTK-associated nodes and downstream branches, whereas sunitinib affected a broader kinase spectrum, consistent with its multi-kinase profile (Figure 1G and Supplemental Figure 1D). Integration of proteomic and phosphoproteomic datasets onto a canonical FLT3 pathway map associated XY0206 treatment with reduced MYC-associated signaling, a key downstream program controlling leukemic cell proliferation, differentiation, and survival (Figure 1H). Immunoblot assays confirmed that XY0206 more effectively suppressed phosphorylation of canonical FLT3 downstream effectors, including STAT5, AKT, and ERK1/2, compared with sunitinib at equivalent concentrations (Figure 1I). Collectively, these data support a model in which XY0206 preferentially suppresses FLT3-centered signaling and downstream antileukemic programs in AML cells.

### XY0206 exhibits preferential antileukemic activity in FLT3-ITD–mutated AML cells.

We next systematically evaluated the antileukemic activity of XY0206 across a panel of AML and *FLT3*-driven cell models. Based on DepMap transcriptomic data and validation by immunoblotting (Supplemental Figure 2, A and B), we selected human AML cell lines with relatively high *FLT3* expression (OCI-AML2), very low *FLT3* expression (HEL), BCR-ABL–driven K562 cells as an *FLT3*-independent kinase-driven control, and *FLT3*-ITD mutations (MV4-11 and MOLM13), as well as murine Ba/F3 cells lacking endogenous *FLT3* expression and Ba/F3 cells engineered to express *FLT3*-ITD. Dose response analyses were performed to determine IC_50_ values, measured using cell viability and proliferation assays, for XY0206, its parental compound sunitinib, and clinically used or approved FLT3is, including gilteritinib, midostaurin, and quizartinib/AC220.

Across the tested models, XY0206 exhibited growth-inhibitory activity comparable to that of approved FLT3is in *FLT3*-ITD-mutated cells. XY0206 suppressed the growth of *FLT3*-ITD-mutated cell lines, including MV4-11, MOLM13, and Ba/F3-*FLT3*-ITD, with IC_50_ values of 5.88 nM, 14.97 nM, and 4.35 nM, respectively. In contrast, higher IC_50_ values were observed in *FLT3*–wild-type (*FLT3*–WT) or *FLT3*-low– or negative-expressing cell lines, including OCI-AML2, HEL, parental Ba/F3 cells, and K562 cells (Table 2 and Supplemental Figure 2C). Together, these findings are consistent with preferential growth-inhibitory activity of XY0206 in *FLT3*-ITD–driven leukemia models, and underscore the influence of *FLT3* expression status in non–*FLT3*-dependent cellular contexts.

We next assessed the functional consequences of XY0206 treatment in *FLT3*-ITD–mutated cells. Exposure to XY0206 for 24 hours resulted in cell-cycle perturbation, characterized by an increased proportion of cells in the G_0_/G_1_ phase (Figure 2A and Supplemental Figure 2, D–F). Apoptosis assays further demonstrated a dose-dependent increase in annexin V–positive cells (Figure 2B and Supplemental Figure 2, G and H), consistent with induction of apoptotic cell death. In line with these phenotypic changes, XY0206 treatment was associated with concentration-dependent suppression of FLT3 and downstream signaling, as evidenced by reduced phosphorylation of FLT3, STAT5, AKT, and ERK1/2. The inhibitory effects of XY0206 on these phosphorylation events were comparable to those observed with gilteritinib and were more pronounced than those induced by sunitinib at matched concentrations. Notably, the reduction in mTOR phosphorylation was less prominent than the inhibition of phosphorylated FLT3, STAT5, AKT, and ERK1/2, arguing against broad suppression of downstream kinase pathways as the primary mechanism of XY0206 activity (Figure 2C). Furthermore, in *FLT3*-independent, BCR-ABL–driven K562 cells, XY0206 at the same concentrations did not suppress phosphorylation of FLT3, STAT5, AKT, ERK1/2, or mTOR (Supplemental Figure 2I). These results support the interpretation that XY0206 suppresses STAT5, AKT, and ERK1/2 signaling primarily through inhibition of mutant FLT3 rather than through direct off-target inhibition of these downstream signaling nodes.

To assess target engagement in intact cells, we performed a cellular thermal shift assay (CETSA) (29). XY0206 treatment increased the thermal stability of FLT3 relative to DMSO control, resulting in a rightward shift of the FLT3 protein melting curve (Figure 2D). This stabilization effect increased with escalating concentrations of XY0206 (0–1,000 nM) (Figure 2E), supporting direct engagement of FLT3 by XY0206 in a cellular context. Collectively, these results indicate that XY0206 directly engages FLT3 and preferentially suppresses *FLT3*-ITD–mutated AML cells through inhibition of FLT3-dependent signaling pathways.

### XY0206 retains activity against FLT3-ITD AML cells harboring the FLT3-F691L gatekeeper mutation.

Acquired resistance to FLT3is frequently arises from treatment-emergent mutations within the TKD of *FLT3* (5, 15, 30, 31). To evaluate the activity of XY0206 against clinically relevant TKD variants, we engineered Ba/F3 cells, which lack endogenous *FLT3* expression, to express *FLT3*-ITD alone or in combination with representative TKD mutations (Supplemental Figure 3A). Gilteritinib retained activity against *FLT3*-ITD-D835V/Y and *FLT3*-ITD-Y842C but showed markedly reduced activity in cells expressing *FLT3*-ITD-F691L. In contrast, XY0206 maintained robust growth-inhibitory activity against Ba/F3-*FLT3*-ITD-F691L cells, whereas the parental compound, sunitinib, exhibited intermediate inhibition. The measured IC_50_ values were as follows: XY0206, 16.69 nM; gilteritinib, 382.97 nM; and sunitinib, 30.17 nM (Figure 3A and Supplemental Figure 3B), highlighting the superior potency of XY0206 in this clinically challenging mutant context. Functional assays further demonstrated that XY0206 treatment induced cell-cycle perturbation and increased apoptosis in Ba/F3-*FLT3*-ITD-F691L cells, effects that were more pronounced than those observed with gilteritinib or sunitinib under matched conditions (Figure 3, B and C, and Supplemental Figure 3, C and D). Immunoblot analysis confirmed that XY0206 effectively reduced phosphorylation of FLT3 and downstream signaling effectors, including STAT5, AKT, and ERK1/2, whereas gilteritinib and sunitinib showed only limited inhibition of these pathways (Figure 3D).

To elucidate the structural basis underlying this activity, we performed molecular docking and molecular dynamics simulations of XY0206 bound to both FLT3-WT and the F691L mutant (FLT3-F691L). Analyses of root mean square deviation, root mean square fluctuation, radius of gyration, and hydrogen-bonding dynamics indicated stable protein–ligand interactions over the simulation period (Supplemental Figure 3, E–H). Molecular mechanics/Poisson–Boltzmann surface area (MM-PBSA) calculations showed binding free energies of –52.894 kcal/mol for FLT3-WT and –60.144 kcal/mol for FLT3-F691L during the final 50 ns of equilibration, suggesting comparable binding affinity of XY0206 for both forms of FLT3. Molecular surface and close-up analyses revealed that XY0206 forms hydrogen bonds with key residues, including Leu616, Glu692, and Asp698, within the FLT3 active site and engages in hydrophobic interactions with Val624, Val675, Leu818, and Phe830. Although substitution of Phe691 with Leu alters 1 hydrophobic interaction, XY0206 maintains multiple stabilizing contacts, including hydrogen bonding with Asp698 and Arg834 and hydrophobic interactions with Val624, Ala642, Val675, Leu818, and Phe830 (Figure 3E).

To experimentally validate these predictions, CETSAs were performed. XY0206 treatment increased the thermal stability of FLT3-ITD-F691L compared with DMSO-treated controls, as reflected by a rightward shift in the protein melting curve (Figure 3F). No appreciable difference in FLT3 stabilization was observed between *FLT3*-ITD cells with or without the F691L mutation, indicating that this substitution does not substantially alter XY0206 binding stability in cells (Figure 3G). In contrast, gilteritinib induced only modest thermal stabilization, whereas XY0206 produced a concentration-dependent increase in FLT3 stability (Figure 3H).

### XY0206 retains activity in FLT3-ITD AML with growth factor–associated and acquired resistance.

Elevated levels of growth factors and cytokines have been implicated in attenuating the response of *FLT3*-ITD AML cells to FLT3is (13, 15). To examine drug activity under such conditions, MOLM13 and MV4-11 cells were treated with increasing concentrations of XY0206, gilteritinib, or sunitinib in the presence or absence of FGF2, which has been used as a simplified, growth factor–based model to assess cytokine-associated modulation of drug response. In cell viability assays, exposure to FGF2 reduced the sensitivity of both cell lines to gilteritinib and sunitinib, resulting in a modest rightward shift of the dose-response curve. In contrast, XY0206 displayed a largely overlapping dose-response profile under FGF2 stimulation, without an evident rightward shift (Figure 4A). Consistent results were observed in cell proliferation assays, in which treatment with XY0206 at its IC_50_ continued to suppress the proliferation of FGF2-stimulated *FLT3*-ITD AML cells, whereas gilteritinib and sunitinib exhibited reduced efficacy under the same conditions (Figure 4B). At the signaling level, immunoblot analysis indicated that FGF2 partially attenuated gilteritinib- and sunitinib-mediated inhibition of phosphorylated FLT3 (p-FLT3) and p-ERK1/2, whereas suppression of these signaling components by XY0206 was preserved (Figure 4C). These findings suggest that XY0206 retains antileukemic activity under growth factor–enriched conditions that diminish the response to gilteritinib and sunitinib.

We next established a cellular model of acquired resistance induced by prolonged exposure to an FLT3i to evaluate the activity of XY0206. MOLM13 cells were continuously cultured in low-dose gilteritinib for approximately 10 months, leading to the establishment of a polyclonal gilteritinib-tolerant subline, designated GilR. Compared with parental MOLM13 cells, GilR cells had a 21-fold increase in gilteritinib IC_50_, from 14.58 nM to 308.70 nM (Figure 4D). Whole-exome sequencing (WES) revealed the accumulation of multiple genetic alterations in GilR cells relative to the parental line, including mutations in genes previously associated with AML progression and drug response, such as *FLT3*, *CCND3*, *CHD7*, *CBL*, *ARID1A*, and *IRS2* (Figure 4E and Supplemental Table 5). Among these alterations, *FLT3* mutations were prominently represented by the F691L gatekeeper substitution, consistent with continued FLT3 pathway engagement in this resistant model. Despite reduced sensitivity to gilteritinib, XY0206 remained active in GilR cells. Treatment with XY0206 resulted in sustained suppression of cell viability (Figure 4F) and was associated with greater inhibition of cell proliferation (Figure 4G), increased accumulation of cells in the G_0_/G_1_ phase, and higher levels of apoptosis compared with gilteritinib at matched concentrations. The effects of sunitinib were intermediate, showing stronger cytotoxicity than gilteritinib but weaker than XY0206 (Figure 4, H and I, and Supplemental Figure 4, A and B). Immunoblot analysis confirmed that XY0206 more completely suppressed phosphorylation of FLT3 and downstream effectors, including STAT5, AKT, and ERK1/2, whereas these pathways remained partially active after gilteritinib or sunitinib treatment (Figure 4J).

Consistent with emerging evidence, a recent conference abstract suggested that *FLT3*-F691L may sustain MAPK signaling through a GRB2-dependent, kinase-independent mechanism under FLT3i treatment (32). To explore whether GRB2-associated signaling might be relevant to XY0206 activity, we examined GRB2 expression in *FLT3*-F691L–resistant models. XY0206 treatment reduced total GRB2 protein levels in both GilR and Ba/F3-*FLT3*-ITD-F691L cells (Supplemental Figure 4, C and D). Moreover, GRB2 depletion further decreased the IC_50_ of XY0206 in GilR cells and enhanced XY0206-mediated killing of resistant cells (Supplemental Figure 4, E and F). These data indicate that GRB2-associated signaling may contribute to the maintenance of *FLT3*-F691L–driven resistant states and can modulate cellular responsiveness to FLT3 inhibition by XY0206. However, these findings do not establish GRB2 as a direct molecular target of XY0206, nor do they support direct disruption of FLT3-GRB2 interaction or kinase-independent FLT3 signaling. Therefore, GRB2 is interpreted here as a functional component of the *FLT3*-F691L–associated signaling network rather than a direct pharmacological target of XY0206.

To further examine whether resistance mechanisms downstream of RTK signaling could limit XY0206 activity, we generated a second long-term, low-dose, gilteritinib-resistant MOLM13 subline, designated GilR2. The IC_50_ for gilteritinib increased from 14.58 nM to 1,174.00 nM (Supplemental Figure 4G). WES analysis revealed, in addition to *FLT3* mutations, a clinically relevant *NRAS* mutation associated with FLT3i resistance (Supplemental Figure 4H and Supplemental Table 5). Drug testing in GilR2 indicated that XY0206 also exhibited reduced activity (Supplemental Figure 4I), suggesting that *NRAS*-mutant subclones may bypass upstream FLT3/RTK-associated signaling and thereby diminish sensitivity to XY0206. These findings further support the presence of heterogeneous resistance mechanisms and indicate that XY0206 activity is most evident in *FLT3*-dependent resistance contexts, whereas resistance driven by downstream RAS pathway activation may require additional therapeutic strategies.

Overall, these data indicate that XY0206 retains activity in *FLT3*-dependent AML models exhibiting reduced responsiveness to gilteritinib, including both growth factor–associated and treatment-emergent resistant contexts, while revealing the impact of multiple resistance mechanisms and subclonal heterogeneity on drug sensitivity.

### XY0206 demonstrates antileukemic activity with favorable tolerability in patient-derived AML blasts and preclinical mouse models.

We next evaluated the activity of XY0206 in patient-derived AML blasts harboring *FLT3*-ITD mutations. Two independent patient samples were analyzed. AML01 was obtained from a patient with relapsed AML refractory to multiple prior therapies, including gilteritinib, whereas AML02 was derived from an unrelated patient with *FLT3*-ITD AML. Targeted sequencing was performed to provide molecular context for these samples. In addition to *FLT3*-ITD, AML01 harbored co-occurring mutations in *DNMT3A*, *IDH2*, and *CEBPA*, and AML02 carried multiple additional AML-associated alterations, including mutations in *WT1*, *TET2*, and *SETDB1* (Supplemental Table 6), highlighting the genetic heterogeneity characteristic of *FLT3*-ITD AML. Across both samples, cell viability assays, direct cell counting, and apoptosis analyses consistently showed that XY0206 produced stronger cytotoxic effects than clinically used FLT3is, including sorafenib and gilteritinib (Figure 5, A–C, and Supplemental Figure 5). Consistent with these functional results, immunoblot analysis demonstrated suppression of p-FLT3 and downstream effectors STAT5, AKT, and ERK1/2, supporting effective inhibition of FLT3-associated signaling in primary AML blasts (Figure 5D).

To assess potential effects on normal hematopoietic cells, cord blood mononuclear cells (CBMCs) from healthy donors were exposed to increasing concentrations of XY0206. CBMC viability remained largely preserved across the tested concentration range, indicating selectivity of XY0206 for leukemic blasts over normal hematopoietic cells (Figure 5E). Antileukemic activity was further examined in vivo using a disseminated leukemia model established by intravenous injection of luciferase-labeled GilR AML cells into NOD.Cg-*Prkdc*^scid^ *Il2rg*^tm1Wjl/SzJ^ (NSG) mice (Figure 5F). XY0206 treatment led to a marked reduction in leukemia burden compared with vehicle, gilteritinib, and sunitinib, as assessed by bioluminescence imaging (Figure 5, G and H). Throughout the treatment period, body weight remained stable and comparable between XY0206-treated and vehicle-treated mice (Figure 5I), suggesting a favorable tolerability profile. In a parallel survival analysis, XY0206 treatment also prolonged mouse survival compared with gilteritinib or sunitinib treatment (Figure 5J). Together, these data demonstrate that XY0206 exhibits potent antileukemic activity in primary *FLT3*-ITD AML blasts and improves leukemia control and survival in preclinical resistant-AML models.

### Phase I/II clinical evaluation of XY0206 monotherapy in patients with R/R AML.

Building on the preclinical activity observed in cellular and mouse models, a phase I/II clinical study was conducted to evaluate the safety, tolerability, pharmacokinetics, and preliminary antileukemic activity of XY0206 in adult patients with R/R AML. The study included an initial dose-escalation stage followed by dose expansion. Patients received continuous oral XY0206 monotherapy until unacceptable toxicity, disease progression, relapse, death, voluntary withdrawal, or transition to subsequent therapy. A total of 35 eligible patients with available genomic profiling were included in the analysis (Figure 6A and Supplemental Figure 6). The median age was 49 years (range, 20–68). This was a heavily pretreated cohort: 33 patients (94.3%) had received anthracycline-containing induction or salvage regimens before enrollment, and 10 patients (28.6%) had prior exposure to FLT3i-based therapy, predominantly sorafenib-based treatment. One patient had received both sorafenib and gilteritinib, whereas 25 patients (71.4%) were FLT3i naive. Detailed patient-level pretreatment histories are provided in Supplemental Table 7. Genomic profiling revealed that 27 patients (77.1%) harbored *FLT3* mutations, including 23 patients (65.7%) with *FLT3*-ITD alone and 2 patients (5.7%) with *FLT3*-TKD alone; 2 patients (5.7%) carried both *FLT3*-ITD and *FLT3*-TKD mutations, and 8 patients (22.9%) carried *FLT3*-WT (Table 3).

During dose escalation, XY0206 was initially administered at planned once-daily dose levels of 12.5, 25, 50, and 62.5 mg. The 50 mg once-daily dose was associated with reduced tolerability, and 1 patient in this cohort experienced a protocol-defined dose-limiting toxicity of colitis. An alternative 25 mg twice-daily schedule was also explored; however, this schedule did not meaningfully improve the tolerability profile. Therefore, an intermediate 37.5 mg once-daily dose level was evaluated to optimize the balance among tolerability, pharmacokinetic exposure, and preliminary antileukemic activity. Pharmacokinetic analyses demonstrated that XY0206 achieved approximately dose-proportional systemic exposure across the evaluated dose range of 12.5 to 62.5 mg. After a single oral dose, the median time to maximum serum concentration (T_max_) ranged from 2.00 to 6.00 hours, with a modest prolongation to 2.00 to 8.00 hours after repeated dosing (Table 4). At steady state, systemic exposure, as assessed by AUC_0–t_, increased by approximately 1.51- to 3.55-fold relative to single-dose administration, and steady-state peak concentrations increased by 1.55- to 3.17-fold, indicating moderate accumulation with repeated dosing (Table 5).

Within this exposure range, XY0206 demonstrated clinically relevant antileukemic activity. According to the 2022 European LeukemiaNet (ELN) response criteria, 2 patients (5.7%) achieved complete remission, 3 patients (8.6%) achieved CRi, and 11 patients (31.4%) achieved an MLFS, resulting in an overall CRc rate, defined as the sum of CR, CRi, and MLFS, of 45.7% (*n* = 16 of 35). Among patients with *FLT3*-ITD mutations, the CRc rate was 60.0% (*n* = 15 of 25). The median duration of response for patients achieving CRc was 45.5 days (range, 10–448 days). The median duration of XY0206 therapy across the full cohort was 57 days (range, 16–506 days). Patient-level treatment exposure, response duration, reason for treatment discontinuation, subsequent HSCT, and grade ≥3 adverse events are summarized in Supplemental Table 7.

Treatment-emergent adverse events (TEAEs) of grade 3 or higher were predominantly hematologic and consistent with the underlying disease, including leukopenia, neutropenia, thrombocytopenia, and anemia, and prior treatment history. Nonhematologic grade ≥3 events were less frequent and included infectious pneumonia, hypokalemia, hypertension, hypertriglyceridemia, rash, bone pain, diarrhea, and colitis. Detailed safety data by dose group are provided in Table 6 and Supplemental Table 8. Reasons for treatment discontinuation included lack of response, relapse after initial response, TEAEs, transition to HSCT, loss to follow-up, voluntary withdrawal, and poor compliance. Achievement of MLFS did not require protocol-mandated interruption or dose reduction of XY0206; treatment continued unless predefined discontinuation criteria were met or patients proceeded to subsequent therapy.

Consistent with these clinical responses, reductions in bone marrow blast percentages were observed in a subset of patients with R/R AML, independent of prior chemotherapy exposure (Figure 6, D and E, and Supplemental Figure 7). Because this phase I/II trial enrolled a broader R/R AML population rather than specifically selecting patients with prior FLT3i exposure, prior FLT3i use was present only in a subset of patients and reflected heterogeneous pre-enrollment treatment histories. Although 10 patients had prior exposure to FLT3i-based therapies, 1 had *FLT3*-TKD mutation without *FLT3*-ITD at enrollment and 1 discontinued XY0206 before completing the first treatment cycle. Thus, 8 patients with *FLT*3-ITD mutations who had received at least 1 prior cycle of FLT3i-based therapy were included in the post-FLT3i efficacy analysis. Among these 8 patients, 3 achieved CRc following XY0206 treatment, and 2 of these patients subsequently proceeded to HSCT (Figure 6, B–E). These observations suggest that XY0206 can achieve sustained systemic exposure and meaningful antileukemic activity in heavily pretreated AML, including the evaluable post-FLT3i *FLT3*-ITD subgroup.

## Discussion

FLT3i resistance in AML is increasingly understood not as a uniform loss of target dependence but as a collection of adaptive disease states in which residual FLT3 signaling continues to support leukemic survival and proliferation (11, 13). This conceptual framework is supported by phosphoproteomic studies in primary AML samples demonstrating that FLT3i response is governed by dynamic kinase signaling states rather than static genetic classification, particularly involving MAPK/ERK pathway activation (33). In this study, we demonstrate that XY0206 targets multiple such FLT3-dependent resistance contexts, providing a conceptual and mechanistic advance beyond the binary classification of FLT3i sensitivity versus resistance.

We show that XY0206, a structurally optimized derivative of sunitinib, exerts a distinct and therapeutically relevant pattern of FLT3 pathway inhibition. Together with biochemical kinase profiling, integrated proteomic and phosphoproteomic analyses support suppression of FLT3-centered signaling networks, with consistent inhibition of canonical downstream pathways including STAT5, AKT, and ERK, accompanied by attenuation of MYC-associated transcriptional programs that are central to FLT3-driven leukemic growth. Structural modeling and CETSA further corroborate direct and stable engagement of FLT3, providing a mechanistic framework linking target binding to downstream pathway suppression.

A key finding of this work is that XY0206 retains antileukemic activity across multiple FLT3-dependent resistance states. In cellular models, XY0206 remained active against *FLT3*-ITD AML cells harboring the F691L gatekeeper substitution, a stringent and well-characterized model of on-target resistance to currently approved FLT3is. Although F691L represents only 1 component of the broader resistance landscape, its structural and biochemical features make it a useful benchmark for evaluating the resilience of FLT3i-target interactions. The preserved activity of XY0206 in this setting underscores its ability to sustain FLT3 pathway inhibition under conditions in which existing inhibitors lose efficacy.

Beyond mutation-associated resistance, XY0206 also demonstrated preserved activity under growth factor–mediated resistance and in models of reduced sensitivity arising from prolonged FLT3i exposure. In contrast to gilteritinib, XY0206 maintained suppression of FLT3 signaling and downstream proliferative pathways in the presence of FGF2 and in gilteritinib-tolerant AML cells. Notably, the activity of XY0206 was most evident in resistant models that retained FLT3-centered signaling dependence, whereas the independently generated GilR2 model, which acquired a clinically relevant *NRAS* mutation, had reduced sensitivity to XY0206. These findings indicate that downstream RAS pathway activation can diminish dependence on upstream FLT3/RTK signaling and may limit the efficacy of XY0206 in FLT3-independent resistance states. Collectively, these models converge on a common biological theme: XY0206 remains effective in leukemic states that continue to rely, at least in part, on FLT3 signaling despite adaptive or acquired resistance to prior FLT3-directed therapy. These observations support the view that FLT3-dependent resistance encompasses a spectrum of biological contexts, rather than a single dominant mechanism.

The clinical observations reported here provide early translational support for these mechanistic findings. In a phase I/II study of patients with relapsed or refractory AML, XY0206 achieved clinically meaningful responses, particularly among patients with *FLT3*-ITD mutations. Responses were also observed in patients previously treated with FLT3is, including individuals who subsequently proceeded to HSCT. Importantly, clinical activity was not restricted to narrowly define genetic-resistance subgroups, consistent with the heterogeneity of FLT3 dependence observed across experimental models. In vitro, XY0206 exhibited nanomolar potency in *FLT3*-ITD–mutated cell lines, intermediate activity in *FLT3*-WT cells with relatively high *FLT3* expression, and minimal activity in cells lacking detectable *FLT3*. Together, these findings support a graded model of *FLT3* dependency in AML, rather than a strictly mutation-based framework. Nevertheless, responses in this heavily pretreated R/R AML cohort were heterogeneous and often limited in duration. Therefore, future clinical development should focus on disease contexts most likely to retain *FLT3* dependence, particularly *FLT3*-ITD R/R AML and patients with reduced sensitivity or resistance to existing FLT3is.

Several limitations of this study merit consideration. First, the availability of viable *FLT3*-F691L–positive primary AML samples was limited, precluding direct ex vivo testing of XY0206 in this specific patient population; complementary *FLT3*-F691L models were used, therefore. Second, resistance driven by *FLT3*-independent pathways was not exhaustively investigated, although the GilR2/*NRAS*-mutant subclone demonstrated reduced XY0206 sensitivity, highlighting the potential influence of downstream RAS pathway activation. Third, we did not directly assess the therapeutic window of XY0206 in a competitive in vivo setting that simultaneously evaluates suppression of *FLT3*-driven leukemia and preservation of normal hematopoietic reconstitution, which will be an important future direction. Finally, although pharmacokinetic analyses demonstrate sustained systemic exposure, direct pharmacodynamic assessment of FLT3 inhibition in patient samples remains limited. Future studies integrating longitudinal molecular profiling and biomarker analyses will be important to refine patient selection strategies and to better define exposure-response relationships.

In summary, this study identifies XY0206 as a structurally and mechanistically differentiated FLT3i that sustains FLT3 pathway inhibition across multiple resistance-associated disease contexts. Rather than aiming to overcome all forms of FLT3i resistance, XY0206 is best positioned for a subset of clinically relevant FLT3-dependent resistance states that remain inadequately controlled by existing therapies. Through integration of biochemical profiling, multi-omics analysis, functional modeling, and early-phase clinical evaluation, our findings establish a coherent mechanistic and translational framework supporting the therapeutic potential of XY0206 in *FLT3*-mutated AML. Ongoing randomized clinical studies, including the phase III trial (ClinicalTrials.gov identifier NCT05876832), will be necessary to define the clinical benefit of XY0206 relative to approved FLT3is and to identify molecular correlates of response and resistance in larger patient populations.

## Methods

### Sex as a biological variable.

Our study examined male and female human patients and animals. Similar findings are reported for both sexes.

### Kinase profiling.

The kinase inhibition profile of XY0206 was determined using a commercial service provided by ICE Bioscience Inc. XY0206 was tested at 100 nM against a panel of 217 human kinases. The assay measured enzymatic activity relative to vehicle controls, and results were reported as percent inhibition for each kinase. Details of the assay workflow, controls, and kinase panel composition were provided by ICE Bioscience.

### Cell lines and cell culture.

MV4-11, MOLM13, HEL, OCI-AML2, K562, and HEK293T cells were provided by Jianxiang Wang (Institute of Hematology & Blood Diseases Hospital, Chinese Academy of Medical Sciences & Peking Union Medical College, Tianjin, China). Ba/F3 cells were provided by Peng Xu (Soochow University, Suzhou, China). MV4-11, MOLM13, and HEL cells were cultured in RPMI-1640 medium (Gibco) supplemented with 10% FBS (Gibco) and 1% penicillin-streptomycin (P/S). OCI-AML2 cells were cultured in MEM Alpha medium (Gibco), K562 cells in IMDM medium (Gibco), and HEK293T cells in DMEM (Gibco), each supplemented with 10% FBS and 1% P/S. GilR and GilR2 cells were generated in-house from parental MOLM13 cells by continuous low-dose gilteritinib exposure until gilteritinib IC_50_ increased by at least 20-fold. No single-cell cloning was performed; therefore, GilR and GilR2 represent polyclonal drug-tolerant populations. GilR cells expressing sgSC-EGFP, sgGRB2-1-EGFP, sgGRB2-2-EGFP, or sgGRB2-3-EGFP were generated by lentiviral infection. Luciferase-expressing MOLM13 cells were generated by lentiviral transduction followed by G418 selection and validation of luciferase expression and activity. Ba/F3 cells were cultured in RPMI-1640 medium supplemented with 10% FBS, 1% P/S, and 3 ng/mL mouse IL-3 (ABclonal, RP01386). Ba/F3-*FLT3*-ITD cells with or without F691L, D835V, D835Y, and Y842C were generated by lentiviral infection with the corresponding *FLT3* constructs. Cells were cultured at 37°C under 5% CO_2_ and were routinely tested and confirmed to be mycoplasma-free.

### Chemicals and reagents.

XY0206 (*R*,*Z*)-*N*-(5-((5-fluoro-2-oxoindolin-3-ylidene)methyl)-2,4-dimethyl-1H-pyrrol-3-yl)pyrrolidine-2-carboxamide hydrochloride was provided by Shijiazhuang Yiling Pharmaceutical Co. Sunitinib (HY-10255A) was purchased from MedChemExpress. Gilteritinib (SJ-MX0187), midostaurin (PKC421), quizartinib/AC220 (SJ-MX0304), and sorafenib (SJ-MX0071A) were purchased from Sparkjade. For in vivo experiments, gilteritinib, sunitinib, and XY0206 were dissolved in a formula containing 50% PEG300 and 50% saline. FGF2 (10014-HNAE) was purchased from SinoBiological.

### Protein extraction and digestion.

Cells were treated with DMSO or XY0206 (10 nM), washed with prechilled PBS by centrifugation (300*g* for 5 minutes), and snap-frozen in liquid nitrogen. Cell pellets were resuspended in buffer containing 1% sodium deoxycholate, 100 mM Tris-HCl, 10 mM tris(2-carboxyethyl)phosphine, and 40 mM chloroacetamide, heated at 95°C for 10 minutes, and sonicated for 2 minutes using a Scientz JY-96-IIN ultrasonicator at 45 W with 3-second on/off cycles. Protein concentration was measured using the Micro BCA kit (Thermo Fisher Scientific, 320058C). For each sample, 200 μg of protein was diluted with 100 mM Tris-HCl to 1 μg/μL and digested with trypsin at a 1:30 enzyme-to-protein ratio at 37°C for 14 hours. Digestion was stopped by adding formic acid (FA) to a final concentration of 1% (v/v). Peptides were desalted using StageTips; 500 ng was used for global proteome analysis, and the remaining peptides were used for phosphopeptide enrichment. Peptides for phosphopeptide enrichment were eluted with loading buffer containing 65% acetonitrile (ACN), 2% trifluoroacetic acid (TFA), and 1 mM KH_2_PO_4_.

### Liquid chromatography–tandem mass spectrometry analysis.

Global proteome analysis was performed using an Orbitrap Exploris 480 mass spectrometer coupled to a FAIMS Pro interface and a Vanquish Neo UHPLC system (Thermo Fisher Scientific). Peptides were loaded onto an Aurora Elite XT C18 UHPLC column (15 cm × 75 μm) and separated using a 51-minute gradient from 3% to 40% solvent B (0.1% FA in 80% ACN) at 200 nL/min. Data-independent acquisition (DIA) included an MS1 scan at 120,000 resolution over a mass/charge ratio (*m*/*z*) of 350–1,250, followed by 22 variable-window MS2 scans at 30,000 resolution. High-field asymmetric waveform ion mobility spectrometry (FAIMS) voltage was set to –45 V, normalized stepped collision energy to 28, and window overlap to 1 *m*/*z*. Global phosphoproteome analysis used the same liquid chromatography–tandem mass spectrometry parameters, except that FAIMS was disabled and the liquid chromatography (LC) gradient was optimized for phosphopeptide separation.

### Global phosphopeptide enrichment.

Phosphopeptide enrichment was performed using titanium dioxide (TiO_2_) beads. Briefly, 1.6 mg of TiO_2_ beads (TiO_2_-to-initial protein amount ratio = 4:1) were equally aliquoted into 2 portions and washed twice with 200 μL of loading buffer (65% ACN, 2% TFA, and 1 mM KH_2_PO_4_). The first aliquot of TiO_2_ beads was added to the eluted peptides and incubated at room temperature for 20 minutes with agitation (1,200 rpm). The supernatant was then transferred to the second aliquot of TiO_2_ beads for an additional 20-minute incubation. After discarding the supernatant, the combined TiO_2_ beads were resuspended in 200 μL of loading buffer, vortexed at 2,000 rpm for 1 minute, and washed 3 times under identical conditions. The beads were transferred to C8 membrane–packed StageTips using 150 μL of loading buffer. After centrifugation, the beads were washed twice with washing buffer (65% ACN, 0.1% TFA) to remove nonspecifically bound peptides. To maximize recovery, phosphopeptides were eluted twice with 50 μL of elution buffer (40% ACN, 15% NH_4_OH). The eluted phosphopeptides were vacuum dried and desalted, then reconstituted in 0.1% FA for subsequent LC–mass spectrometry (LC-MS) analysis.

### Tyrosine phosphopeptide enrichment.

Tyrosine-phosphorylated peptides were enriched using anti-phosphotyrosine antibody-conjugated agarose beads (PTMScan Phospho-Tyrosine Motif Kit; WM1302, Micrometer Biotech Company) according to the manufacturer’s instructions. Briefly, desalted peptide samples were resuspended in IP buffer (pH 7.0–7.5) and subjected to mixing and centrifugation to ensure complete dissolution. Antibody-conjugated beads were equilibrated and washed with PBS prior to incubation with the peptide samples. For each sample, peptides were incubated with beads under gentle agitation overnight at 4°C, followed by sequential washes with IP wash buffers to remove nonspecifically bound peptides. Bound peptides were eluted using 0.2% TFA, pooled, and subjected to vacuum drying. The eluted phosphopeptides were vacuum dried and desalted, then reconstituted in 0.1% FA for subsequent LC-MS analysis.

### Data processing.

Raw DIA files were processed using Spectronaut (Biognosys, v19.7.250203.62635) in directDIA mode against the UniProtKB human reference database (release April 2021; *n* = 20,387 reviewed entries). The entire workflow was performed using default settings unless otherwise specified. The specific parameters were configured as follows: the enzyme was set to trypsin/P, with a maximum of 2 missed cleavages allowed per peptide during the search. Carbamidomethylation of cysteine was defined as a fixed modification; phosphorylation of serine, threonine, and tyrosine (excluded in global proteome database searches), oxidation of methionine, and N-terminal acetylation of proteins were included as variable modifications. The false discovery rate for both protein and peptide precursors was set to 1%.

### Data preprocessing and statistical analysis.

For the global proteome, the data were first subjected to median normalization, followed by protein filtering to retain proteins with valid values in at least 50% of all samples. Missing values were imputed using the k-nearest neighbors algorithm, and protein intensity values were subsequently log_2_ transformed. The resulting matrix, comprising 12 samples and 7,391 proteins, was used for subsequent statistical and bioinformatics analyses. For the phosphoproteome data, median-centered normalization was applied, and the dataset was filtered to retain entries with less than 50% missing values. A log_2_-transformed expression matrix containing 12 samples and 42,202 phosphorylation sites was generated for further analysis. Based on the preprocessed expression matrices, differentially expressed proteins and phosphosites were identified using the limma package (version 3.54.2). Proteins or phosphosites with a fold change > 1.5 and a *P* value < 0.05 were considered statistically significant and classified as differentially expressed.

### Enrichment analysis of pathways.

Pathway enrichment analysis of the differentially expressed proteins and phosphoproteins was performed using the Metascape platform with default parameters (http://metascape.org). Gene Ontology terms with a Benjamini-Hochberg adjusted *P* value < 0.05 were considered statistically significant. Gene Set Enrichment Analysis (GSEA) was conducted using the clusterProfiler package (v4.6.2). Prior to enrichment analysis, phosphosite-level fold changes were aggregated at the corresponding protein-gene level. The canonical pathway gene sets from the Molecular Signatures Database (MSigDB) (c2.cp.v2024.1.Hs.symbols.gmt) were used for the GSEA.

### Kinase activity inference.

Kinase activity was inferred from differentially regulated phosphorylation sites using 2 independent phosphosite-based kinase activity inference platforms, KSEA and RoKAI. Both approaches estimate kinase activity by integrating quantitative changes in phosphorylation of annotated kinase-substrate peptide sequences and do not rely on total kinase or substrate protein abundance. For the KSEA annotation analysis, the “PhosphoSitePlus + NetworKIN” database was used, with thresholds for NetworKIN and *P* value set at 1.5 and 0.05, respectively, and a minimum substrate count of 2. For RoKAI analysis, raw fold changes were used as input, the kinase-substrate database was set to “PSP+SIGNOR,” the RoKAI network was selected as “KS+PPI+SD+CoEv,” and phosphatases were excluded from the analysis. To improve robustness and reduce algorithm-specific bias, kinase activities inferred by KSEA and RoKAI were analyzed independently, and kinases consistently identified by both methods were selected for visualization and downstream interpretation. Kinases common to both KSEA and RoKAI were selected for visualization, which was performed using the KinMap web tool (http://kinhub.org/kinmap/).

### Cell viability assay and cell proliferation analysis.

Human leukemia cells were seeded into 96-well plates at a density of 4,500 cells/well. The cells were then treated with increasing concentrations of the respective drugs. After 3 days, cell viability was evaluated using the Cell Counting Kit-8 reagent (Apexbio, K1018) following the manufacturer’s instructions. The IC_50_ values were determined through dose-response modeling. For cell proliferation analysis, cells were seeded into 12-well plates (2 × 10^5^ cells/well) and treated with XY0206, gilteritinib, sunitinib, or sorafenib. Then cultured cells subjected to different treatments were counted every 2 or 3 days using a hemocytometer.

### Cell apoptosis and cell cycle analysis.

Cells were treated with XY0206, gilteritinib, sunitinib, or sorafenib for 3 days and then washed 3 times with PBS. For the cell apoptosis assay, apoptotic cells were detected using an annexin-V/propidium iodide (PI) apoptosis detection kit (Yeasen, 40302ES60) per the manufacturer’s protocols. For cell-cycle analysis, cells were permeabilized with precooled 70% ethanol at –20°C overnight, and stained in 50 μg/mL PI supplemented with 50 μg/mL DNase-free RNaseA at 37°C for 30 minutes. All samples were determined by an LSRFortessa flow cytometer (BD Biosciences). Data were acquired by flow cytometry and analyzed with FlowJo (Tree Star).

### Protein isolation and Western blot analysis.

Cells were lysed in cold RIPA lysis buffer supplemented with protease and phosphatase inhibitor cocktails. Equal amounts of total protein were separated by SDS-PAGE and analyzed by Western blotting using antibodies against p-FLT3 (Cell Signaling Technology, 60413), p-STAT5A (Sangon Biotech, D155020), p-AKT (Cell Signaling Technology, 9271S), p-ERK1/2 (Cell Signaling Technology, 9101S), p-c-MYC (Abcam, ab185656), p-mTOR (ABclonal, AP0115), FLT3 (Sangon Biotech, D221706), STAT5A (Sangon Biotech, D220085), AKT (Cell Signaling Technology, 9272S), ERK1/2 (Cell Signaling Technology, 9102S), c-MYC (Abcam, ab32072), mTOR (Sangon Biotech, D155337), and GAPDH (Cell Signaling Technology, 5174). Protein bands were visualized using an ECL chemiluminescence kit (Yeasen, 36208ES60).

### Molecular docking and molecular dynamics simulation.

Molecular docking studies were conducted using AutoDock. The X-ray crystal structure of FLT3 was downloaded from the Protein Data Bank (identifier 5X02). The entire docking process was carried out according to the AutoDock User Guide, and the parameters were all the default. After docking, the pose with the highest score was selected, and the binding mode was analyzed and imaged using ChimeraX. Molecular dynamics simulation was performed by GROMACS. The initial protein conformation was parameterized using the AMBER99SB-ILDN force field, and the AMBER force field parameters of the ligand were generated by ACPYPE. A solvated unit cell with water as the solvent was constructed, and the system was neutralized with Na^+^ ions. Energy minimization was then carried out in 2 stages using the steepest descent and conjugate gradient methods to relax the structure and achieve an optimized geometry. Subsequently, equilibration was performed in the NVT and NPT ensembles at a fixed temperature of 300 K for 200 ps each, ensuring system stability before production simulations. A 100 ns molecular dynamics simulation was then performed to investigate the conformational dynamics and binding interactions of the protein-ligand complex. The MM/PBSA calculations were performed using gmx_MMPBSA.

### The CETSA.

For CETSA, cells were treated with DMSO or XY0206 (100 nM) for 1 hour, harvested, and resuspended in prechilled PBS containing PMSF (Solarbio, 329-98-6) and protease-inhibitor cocktail (Beyotime, P1046-1). Samples were aliquoted into PCR tubes, heated at the indicated temperatures for 3 minutes, cooled at 25°C for 3 minutes, and lysed by liquid nitrogen freeze-thawing. Lysates were centrifuged, and supernatants were analyzed by SDS-PAGE and immunoblotting. For isothermal dose-response CETSA, cells were seeded in 6-well plates at 3 × 10^6^ cells/well and treated with increasing concentrations of XY0206 for 30 minutes. Samples were then heated at the indicated temperature for 3 minutes, and cell lysis and protein detection were performed as just described.

### Primary AML samples from patients and cord blood mononuclear cells.

The acquisition of human samples was approved by the Institute of Hematology & Blood Diseases Hospital, Chinese Academy of Medical Sciences & Peking Union Medical College, in compliance with assurances filed with the Department of Health and Human Services (approval CIFMS2025039-EC-2). Primary AML samples and CBMCs were cultured in MethoCult H4434 Classic medium (4434, STEMCELL), supplemented with IL-3 (20 ng/mL; PeproTech), FLT3 ligand (100 ng/mL; PeproTech), TPO (50 ng/mL; PeproTech), SCF (100 ng/mL; PeproTech), 2 mM glutamine, and 1% P/S.

### Animal models.

For in vivo experiments, 6- to 8-week-old NSG mice were obtained from Beijing Vital River Laboratory Animal Technology Co. Luciferase-expressing GilR cells were intravenously injected into NSG mice. The mice were randomly assigned to 4 groups. Starting 6 days after cell injection, tumor-bearing mice were orally treated once daily with XY0206 (20 mg/kg), gilteritinib (20 mg/kg), sunitinib (20 mg/kg), or vehicle control. To evaluate therapeutic efficacy, bioluminescence imaging was performed every 3 or 4 days during the treatment period. Survival was assessed in parallel in an independent cohort under the same treatment schedule. All animal experiments were approved by the Animal Welfare and Ethics Committee of the Institute of Hematology & Blood Diseases Hospital, Chinese Academy of Medical Sciences & Peking Union Medical College (approval IHCAMS-DWLL-CIFMS2025039-2).

### Clinical trials and patient information collection.

The clinical studies were approved by the Institutional Review Board of the Institute of Hematology & Blood Diseases Hospital, Chinese Academy of Medical Sciences & Peking Union Medical College, and were registered with ClinicalTrials.gov. A phase I/II clinical trial (ClinicalTrials.gov identifier NCT04471064) was conducted to evaluate the safety, tolerability, pharmacokinetics, and preliminary efficacy of XY0206 monotherapy in patients with R/R AML. All participants provided written informed consent, and the study was conducted in accordance with the Declaration of Helsinki and institutional review board policies.

Thirty-five patients with R/R AML were enrolled at a single center between September 9, 2020, and April 10, 2024. Eligible patients were aged ≥18 years with a confirmed diagnosis of primary or secondary AML based on bone marrow morphology, an Eastern Cooperative Oncology Group performance status of ≤2, adequate organ function, and an estimated life expectancy of at least 12 weeks. Key exclusion criteria included a diagnosis of acute promyelocytic leukemia, central nervous system involvement, presence of the BCR-ABL fusion gene, New York Heart Association class III/IV heart failure, and uncontrolled active infections, clinically important arrhythmias, angina, or myocardial infarction within the preceding year.

The study consisted of 2 sequential parts: a dose-escalation phase (part 1) followed by a dose-expansion phase (part 2). Part 1 was designed to determine the maximum tolerated dose and/or the recommended phase 2 dose of XY0206. Initiation of part 2 was contingent upon interim assessment of safety, tolerability, pharmacokinetics, and preliminary antileukemic activity observed in part 1. Dose escalation followed a modified Fibonacci design, with planned once-daily dose levels of 12.5, 25, 50, and 62.5 mg. Enrollment began with a single patient at the lowest dose level, followed by cohort expansion according to predefined escalation criteria. Based on emerging safety, pharmacokinetic, and preliminary efficacy data, additional cohorts were introduced, including an alternative 25 mg twice-daily schedule and an intermediate 37.5 mg once-daily dose level.

The primary objective of the study was to evaluate the safety and tolerability of XY0206, and the secondary objective was to assess preliminary antileukemic efficacy. Clinical data collected included patient demographics, prior treatment regimens, mutation profiles, TEAEs, bone marrow blast percentages, and peripheral blood cell counts. Safety assessments were based on TEAEs, hematologic parameters, and treatment exposure. Efficacy was evaluated according to changes in bone marrow blast percentage and peripheral blood cell counts, with responses assessed according to the 2022 ELN response criteria.

Dose-limiting toxicities (DLTs) were defined as adverse events considered at least possibly related to XY0206 and occurring during the first treatment cycle of the dose-escalation phase, with each treatment cycle defined as 28 days. DLTs included any grade ≥4 nonhematologic toxicity and any grade ≥3 nonhematologic toxicity that did not resolve to grade ≤2 within 3 days despite optimal supportive care. Exceptions included grade 3 anorexia, fatigue, infection, febrile neutropenia, medically controlled hypertension, and grade 3 nausea, vomiting, diarrhea, or elevated alanine aminotransferase or aspartate aminotransferase levels, provided that these events improved to grade ≤2 within 7 days with standard medical management. DLTs also included grade ≥2 irreversible nephrotoxicity, cardiotoxicity, or neurotoxicity; grade ≥2 pancreatitis; and grade ≥3 serum amylase or lipase elevation requiring clinical intervention. Hematologic toxicities were not considered DLTs, except for grade 4 neutropenia or thrombocytopenia that failed to recover by day 42 in patients with bone marrow blasts <5%. One DLT, colitis, occurred in the 50 mg once-daily dose cohort.

### Statistics.

Clinical safety endpoints were assessed in the safety set, and antileukemic efficacy was evaluated in the full analysis set, which included all patients who received at least 1 dose of XY0206. The sample size was determined by the phase I/II study design rather than by a formal statistical power calculation; enrollment of 35 patients was considered sufficient for assessment of safety, tolerability, pharmacokinetics, and preliminary antileukemic activity.

Statistical analyses were performed using GraphPad Prism, version 9.0. Comparisons between 2 groups were conducted using 2-tailed unpaired *t* tests. Comparisons among multiple groups were performed using 1-way or 2-way ANOVA, as appropriate. Gene and protein expression levels were normalized to GAPDH, the stability of which was verified across experimental conditions. Quantitative cellular assays were performed with at least 3 independent biological replicates unless otherwise indicated. Immunoblot experiments were repeated independently at least twice with similar results, as specified in the figure legends. Data are presented as mean ± SD. *P* < 0.05 was considered significant.

### Study approval.

All animal experiments were approved by the Animal Welfare and Ethics Committee of the Institute of Hematology & Blood Diseases Hospital, Chinese Academy of Medical Sciences & Peking Union Medical College, Tianjin, China (approval no. IHCAMS-DWLL-CIFMS2025039-2). The acquisition and use of primary human samples were approved by the Institutional Review Board of the Institute of Hematology & Blood Diseases Hospital, Chinese Academy of Medical Sciences & Peking Union Medical College, Tianjin, China (approval no. CIFMS2025039-EC-2). The phase I/II clinical study was approved by the same Institutional Review Board. All participants provided written informed consent.

### Data availability.

The MS proteomics data and Spectronaut database search files have been deposited in the ProteomeXchange Consortium (http://proteomecentral.proteomexchange.org) via the iProX partner repository (34, 35) and are publicly available under dataset identifier PXD061487 at https://www.iprox.cn/page/project.html?id=IPX0011226000 The raw WES data generated in this study have been deposited in the Genome Sequence Archive for Human at the National Genomics Data Center, China National Center for Bioinformation, and are publicly available under accession number HRA020321 (BioProject PRJCA071734) at https://ngdc.cncb.ac.cn/gsa-human/browse/HRA020321.Values for all data points shown in graphs are provided in the Supporting Data Values file.

## Author contributions

HW, TC, L Shen, and JQ conceived the project and designed the experiments. HW, L Shen, and MN wrote and edited the manuscript. YY, CX, JF, NW, YF, and LK conducted the experiments. XD, XS, S Li, S Liu, and DZ performed LC–tandem MS data analysis. BJ, L Song, and MS participated in and executed the clinical trial. CH and YJ conducted the molecular docking experiments and related analysis. All authors approved the submitted version and vouch for the accuracy of the data reported and adherence to the protocol.

## Conflict of interest

The authors have declared that no conflict of interest exists

## Funding support

- Chinese Academy of Medical Sciences Innovation Fund for Medical Sciences (grants 2023-I2M-3-014, 2023-I2M-2-007, 2025-I2M-TS-07, and 2025-I2M-XHJC-037 to HW; 2025-I2M-XHXX-135 to L Shen; and 2025-I2M-XHXX-134 to MN).
- Tianjin Municipal Natural Science Foundation of China (grant 22JCJQJC00090 to HW).
- Tianjin Municipal Science and Technology Commission (grants 25ZXRKSY00050 and 25ZXZSSS00410 to HW).
- National Natural Science Foundation of China (grants 82270236 and 82341080 to HW; 82273217 to L Shen; 82400271 to MN; and 82403710 to LK).
- Haihe Laboratory of Cell Ecosystem Innovation Fund (grant 25HHXBSS00001 to MN).
- Sichuan Science and Technology Program (grant 2025ZNSFSC0737 to HW).
- China Foundation for Youth Entrepreneurship and Employment–Incaier Public Welfare Fund (grants HH25KYHX0016 to JF and HH25KYHX0010 to YY).
- Shijiazhuang Yiling Pharmaceutical Co., Ltd.

## Supplementary Material

Supplemental data

Unedited blot and gel images

Supplemental table 1

Supplemental table 2

Supplemental table 3

Supplemental table 4

Supplemental table 5

Supplemental table 6

Supplemental table 7

Supplemental table 8

Supporting data values

## Figures and Tables

**Figure 1:**
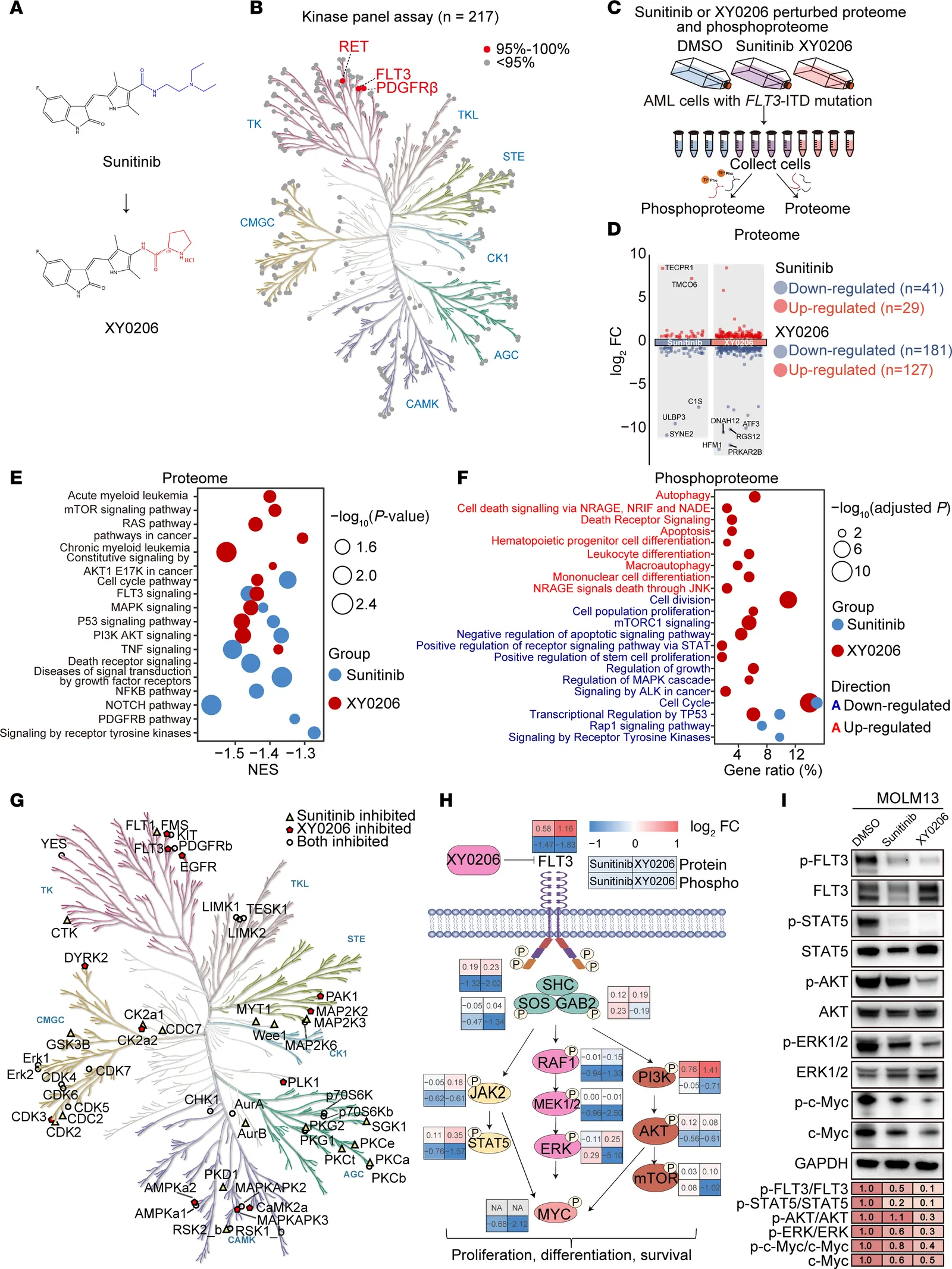
Biochemical and multi-omics profiling define an FLT3-centered inhibitory profile of XY0206. (**A**) Chemical structures of sunitinib and XY0206, with structural differences highlighted in blue and red. (**B**) Kinase inhibition profiling of XY0206 at 100 nM across 217 kinases. (**C**) Workflow for proteomic and phosphoproteomic profiling of *FLT3*-ITD AML cells treated with sunitinib or XY0206. (**D**) Scatterplots of differentially expressed proteins after sunitinib or XY0206 treatment. Red and blue dots indicate upregulated and downregulated proteins, respectively; selected proteins are labeled. (**E**) Pathway enrichment analysis of proteins regulated by XY0206 or sunitinib. NES, normalized enrichment score. (**F**) GSEA of phosphosite alterations after XY0206 or sunitinib treatment. Canonical pathways were obtained from MSigDB. (**G**) Kinase activity inference based on tyrosine-enriched phosphoproteomics using KSEA and RoKAI. Kinases shown were identified by both methods, with *P* < 0.05 and at least 2 substrate sites. Node shapes indicate treatment specificity: triangles, sunitinib-specific; pentagons, XY0206-specific; circles, shared. (**H**) Integrated FLT3 pathway map showing MS-derived protein and phosphosite-level changes after treatment. Heatmap values indicate log_2_ fold changes (FCs) from the corresponding proteomic or phosphoproteomic datasets; they should not be interpreted as phosphorylation (Phospho) occupancy, absolute phosphoprotein abundance, or mathematically combinable values across datasets. (**I**) Immunoblot analysis of total and phosphorylated FLT3, STAT5, AKT, ERK1/2, and c-MYC in MOLM13 cells treated with XY0206 or sunitinib (10 nM) for 3 hours. Numbers below blots indicate densitometric ratios normalized to total protein or GAPDH and scaled to DMSO. Immunoblots were repeated independently at least twice with similar results.

**Figure 2:**
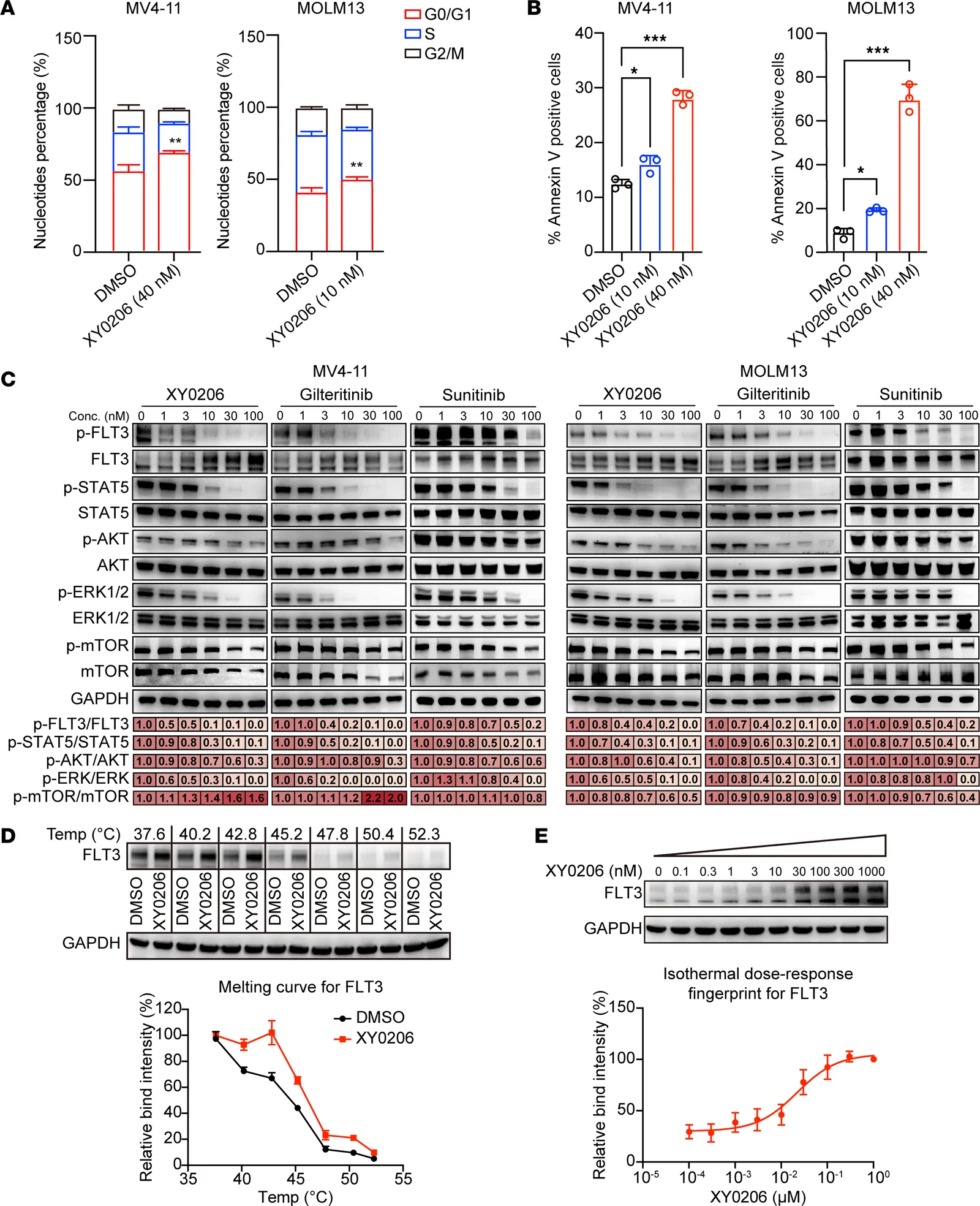
XY0206 exhibits preferential antileukemic activity in *FLT3*-ITD—mutated AML cells. (**A**) Cell cycle distribution of MV4-11 and MOLM13 cells treated with DMSO or XY0206 for 24 hours, assessed by PI staining and flow cytometry. (**B**) Apoptosis of MV4-11 and MOLM13 cells treated with DMSO, 10 nM XY0206, or 40 nM XY0206 for 48 hours, assessed by annexin V/PI staining and flow cytometry. (**C**) Immunoblot analysis of total and phosphorylated FLT3, STAT5, AKT, ERK1/2, and mTOR in *FLT3*-ITD–mutated MV4-11 and MOLM13 cells treated with XY0206, gilteritinib, or sunitinib at the indicated concentrations for 3 hours. Numbers below blots indicate densitometric ratios of phosphorylated to total protein normalized to vehicle-treated controls. (**D**) CETSA analysis of FLT3 thermal stabilization in MOLM13 cells treated with XY0206 (100 nM) or DMSO for 1 hour across the indicated temperature gradient. (**E**) Isothermal dose-response CETSA analysis of FLT3 thermal stabilization in MOLM13 cells treated with increasing concentrations of XY0206 for 1 hour, followed by heating at 50.4°C for 3 minutes. Data are shown as mean ± SD. For **A**, *n* = 3 biological replicates; for **B**, *n* = 3 independent biological experiments. Statistical analyses were performed using 2-tailed unpaired Student’s *t* test for **A** and 1-way ANOVA followed by Dunnett’s test for **B**. *P < 0.05, **P < 0.01, ***P < 0.001. All immunoblots were repeated independently at least twice with similar results, and representative blots are shown.

**Figure 3:**
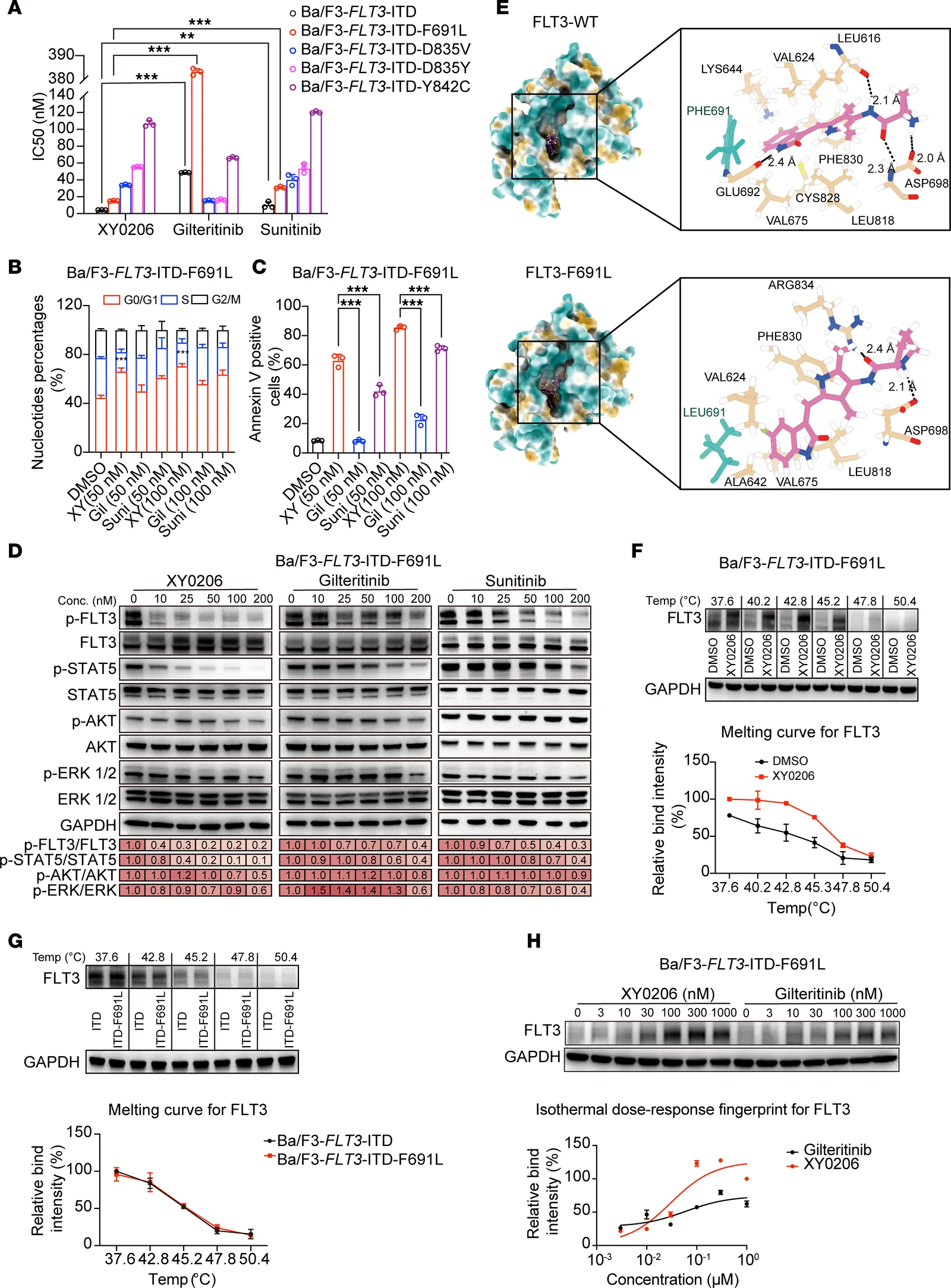
XY0206 retains activity against *FLT3*-ITD AML cells harboring the *FLT3*-F691L gatekeeper mutation. (**A**) IC_50_ values of XY0206, gilteritinib, and sunitinib in Ba/F3-*FLT3*-ITD cells with or without *FLT3*-TKD mutations after 72 hours of treatment. (**B**) Cell-cycle distribution of Ba/F3-*FLT3*-ITD-F691L cells treated with increasing concentrations of XY0206 (XY), gilteritinib (Gil), or sunitinib (Suni) for 24 hours, analyzed by PI staining and flow cytometry; comparisons were performed between XY0206 and gilteritinib at matched concentrations. (**C**) Apoptosis of Ba/F3-*FLT3*-ITD-F691L cells treated with XY0206, gilteritinib, or sunitinib for 48 hours, analyzed by annexin V/PI staining and flow cytometry. (**D**) Immunoblot analysis of total and phosphorylated FLT3, STAT5, AKT, and ERK1/2 in Ba/F3-*FLT3*-ITD-F691L cells treated with XY0206, gilteritinib, or sunitinib for 3 hours. Numbers below blots indicate densitometric ratios of phosphorylated to total protein normalized to vehicle-treated controls. (**E**) Molecular docking analysis of XY0206 bound to FLT3; dashed lines indicate hydrogen bonds. (**F**) CETSA analysis of FLT3 thermal stabilization in Ba/F3-*FLT3*-ITD-F691L cells treated with XY0206 (100 nM) or DMSO across the indicated temperature gradient. (**G**) CETSA analysis of FLT3 thermal stabilization in Ba/F3-*FLT3*-ITD and Ba/F3-*FLT3*-ITD-F691L cells treated with XY0206. (**H**) Isothermal dose-response CETSA analysis of FLT3 thermal stabilization in Ba/F3-*FLT3*-ITD-F691L cells treated with increasing concentrations of XY0206 or gilteritinib for 1 hour, followed by heating at 47.8°C for 3 minutes. Data are shown as mean ± SD from 3 independent biological experiments for **A**–**C**. Statistical analyses were performed using 2-tailed unpaired Student’s *t* test for **A** and 1-way ANOVA followed by Tukey’s multiple-comparison test for **B** and **C**. **P < 0.01, ***P < 0.001. All immunoblots were repeated independently at least twice with similar results, and representative blots are shown.

**Figure 4:**
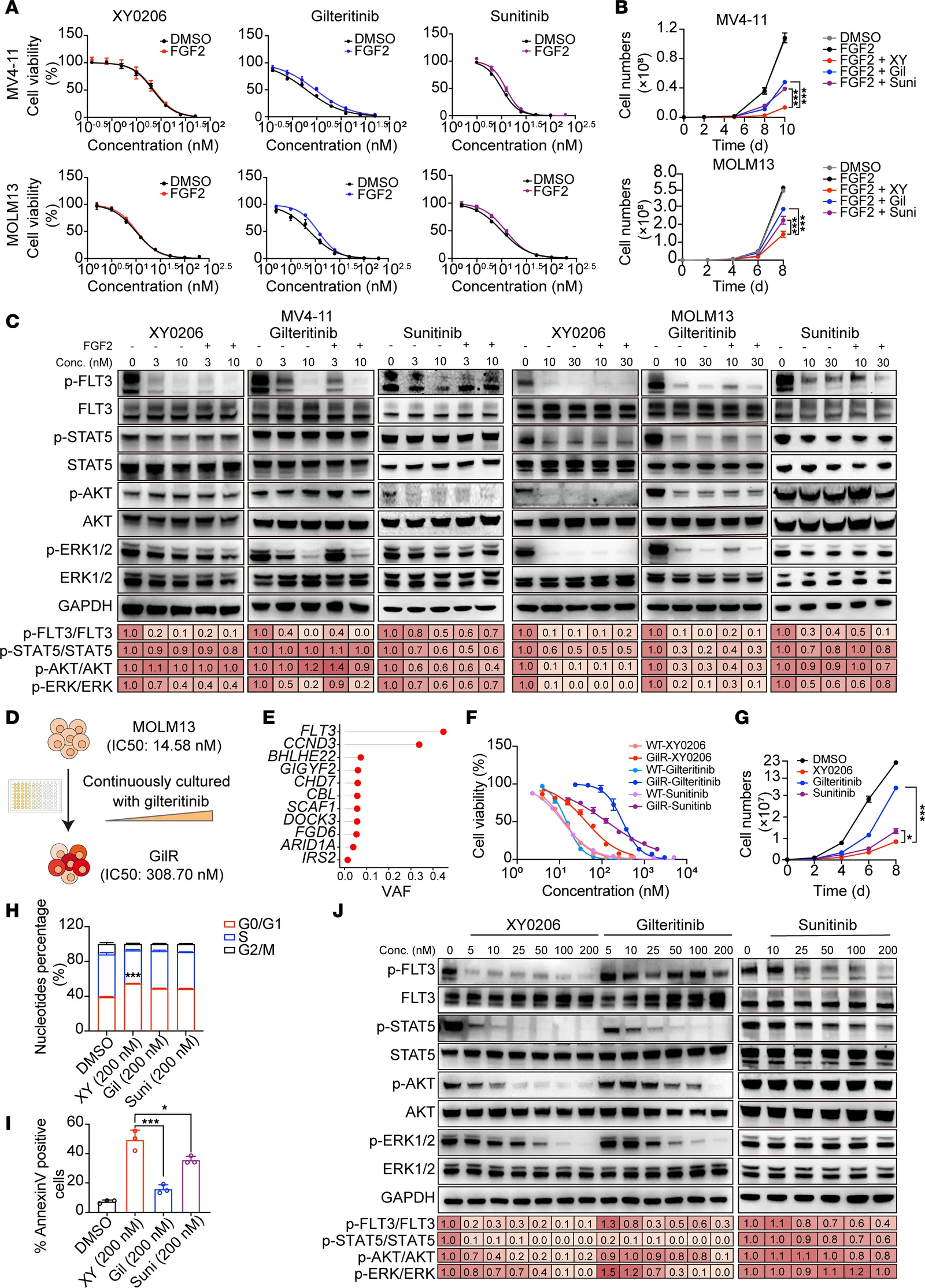
XY0206 retains activity in *FLT3*-ITD AML with growth factor—associated and acquired resistance. (**A**) CCK-8 viability assays of MV4-11 and MOLM13 cells treated with XY0206, gilteritinib, or sunitinib for 3 days with or without FGF2 (10 ng/mL). (**B**) Growth kinetics of MV4-11 and MOLM13 cells cultured with or without FGF2 (20 ng/mL) and treated at respective IC_50_ concentrations. Cells were counted every 2 or 3 days for up to 10 days (MV4-11) or 8 days (MOLM13). (**C**) Immunoblot analysis of phosphorylated and total FLT3, STAT5, AKT, and ERK1/2 in MV4-11 and MOLM13 cells treated with XY0206, gilteritinib, or sunitinib for 10 minutes with or without FGF2. Conc, concentration. (**D**) Schematic of GilR cell generation and gilteritinib IC_50_ determination. (**E**) WES analysis of GilR cells showing acquired genetic alterations relative to MOLM13. VAF, variant allele frequency. (**F**) CCK-8 dose-response curves of parental MOLM13 and GilR cells treated with increasing concentrations of XY0206, gilteritinib, or sunitinib for 72 hours. (**G**) Proliferation of GilR cells treated with XY0206, gilteritinib, or sunitinib (80 nM), measured every 2 days for 8 days. (**H**) Cell cycle distribution of GilR cells treated with XY0206 (XY), gilteritinib (Gil), or sunitinib (Suni) for 24 hours. (**I**) Apoptosis of GilR cells treated with XY0206, gilteritinib, or sunitinib for 72 hours. (**J**) Immunoblot analysis of total and phosphorylated FLT3, STAT5, AKT, and ERK1/2 in GilR cells treated for 4 hours. Numbers below blots in **C** and **J** indicate densitometric ratios normalized to vehicle controls. Data are mean ± SD from 3 independent biological experiments for **A**, **B**, and **F**–**I**. Statistical analyses were performed using 2-way ANOVA with Tukey’s test for **B** and **G** and 1-way ANOVA with Tukey’s test for **H** and **I**. *P < 0.05, ***P < 0.001. Immunoblots in **C** and **J** were repeated independently at least twice with similar results.

**Figure 5:**
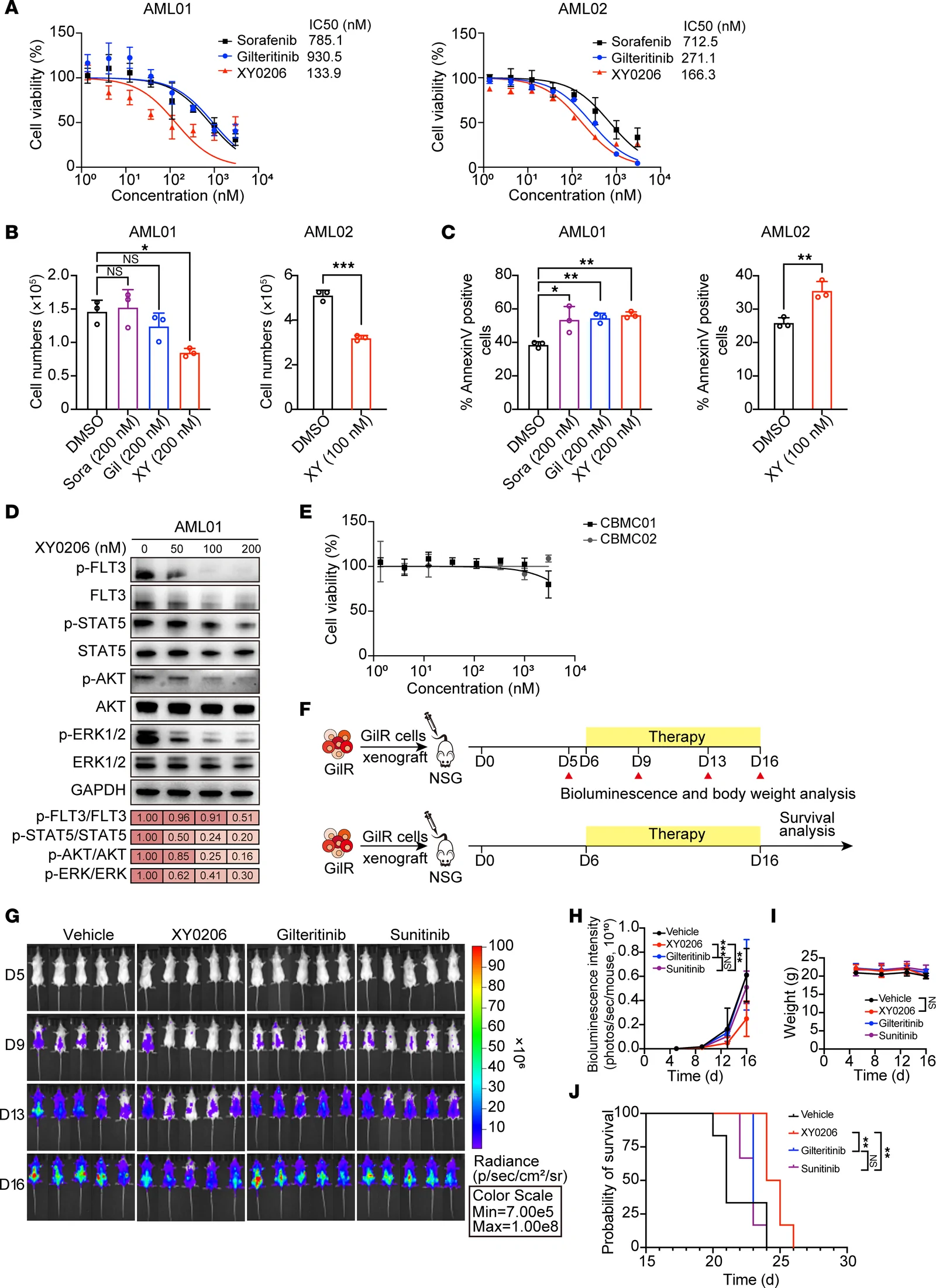
XY0206 demonstrates antileukemic activity with favorable tolerability in patient-derived AML blasts and preclinical mouse models. (**A**) Cell viability of primary *FLT3*-ITD AML blasts (AML01 and AML02) treated with sorafenib, gilteritinib, or XY0206 for 72 hours; IC_50_ values were calculated using GraphPad Prism. (**B**) Viable cell counts of primary AML blasts after 48-hour treatment with sorafenib (Sora), gilteritinib (Gil), or XY0206 (XY). (**C**) Apoptosis of primary AML blasts after 48-hour treatment with sorafenib, gilteritinib, or XY0206, assessed by annexin V/PI staining and flow cytometry. (**D**) Immunoblot analysis of phosphorylated and total FLT3, STAT5, AKT, and ERK1/2 in AML01 cells treated with XY0206 for 24 hours. Numbers below blots indicate densitometric ratios normalized to total protein and scaled to vehicle control. (**E**) Viability of CBMC01 and CBMC02 treated with increasing concentrations of XY0206 for 72 hours. (**F**) Schematic of the cell line-derived xenograft model and treatment schedule. D, day. (**G** and **H**) Representative longitudinal bioluminescence images and quantification of leukemia burden in mice treated with vehicle, XY0206, gilteritinib, or sunitinib. Imaging was performed every 3 or 4 days. (**I**) Body-weight monitoring during treatment. (**J**) Kaplan-Meier survival analysis from an independent cohort treated with the same dosing schedule. *n* = 6 mice per group. Data are mean ± SD from 3 independent experiments for **A**–**C** and **E**, and from 5 mice/group for **H** and **I**. For **B** and **C**, multigroup comparisons were analyzed by 1-way ANOVA with Tukey’s test and 2-group comparisons by 2-tailed unpaired Student’s *t* test. For data reported in **H** and **I**, 2-way ANOVA with Tukey’s test was used. For data reported in **J**, a log-rank test was used. *P < 0.05, **P < 0.01, ***P < 0.001. Immunoblots in **D** were repeated independently at least twice with similar results.

**Figure 6:**
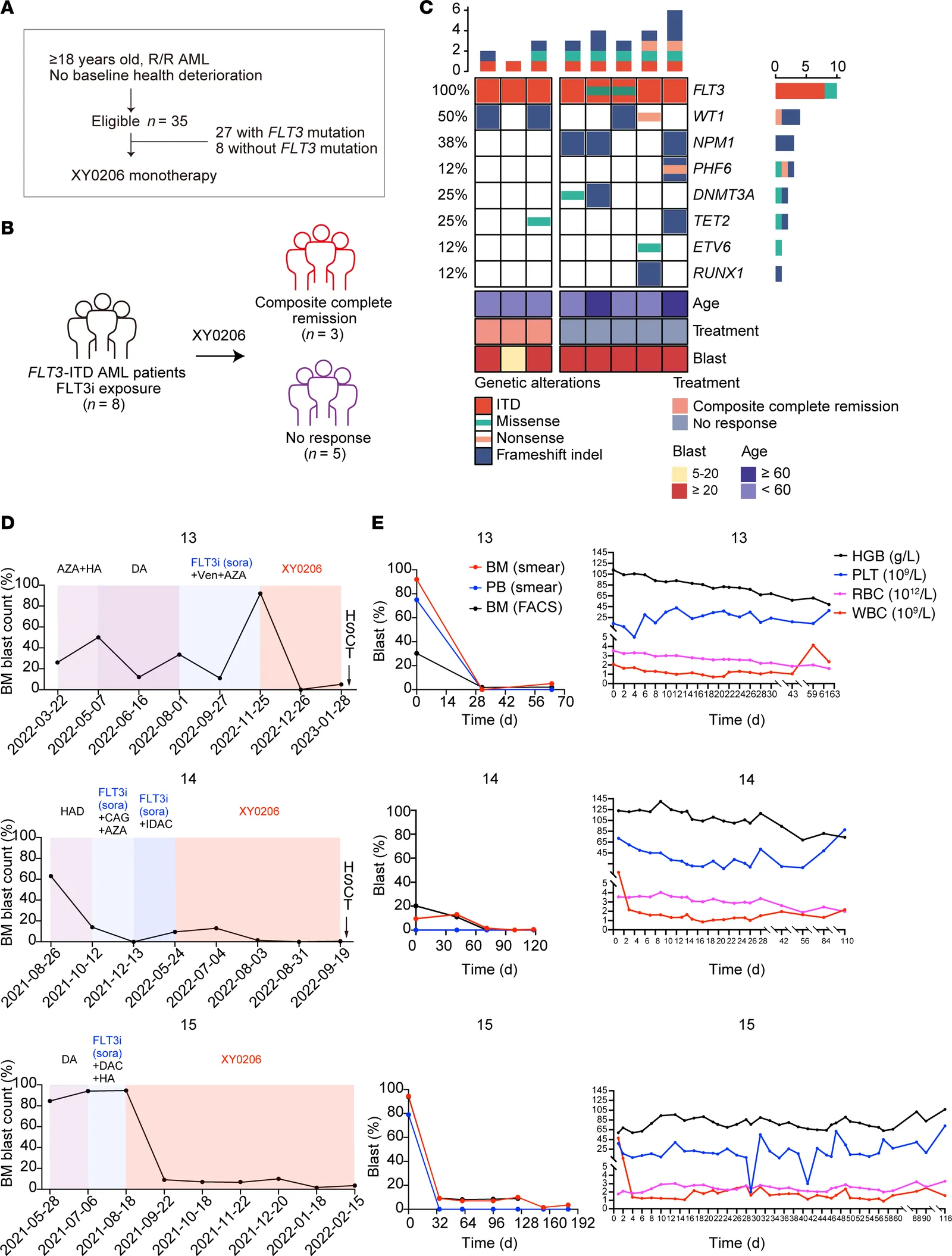
Phase I/II clinical evaluation of XY0206 monotherapy in patients with R/R AML. (**A**) Consolidated Standards of Reporting Trials (CONSORT) diagram of the single-center phase I/II trial. (**B**) Overview of treatment efficacy in patients with FLT3i-exposed R/R AML who received XY0206 monotherapy, with a median treatment duration of 34 days. (**C**) Baseline clinical and molecular characteristics of patients with FLT3i-exposed R/R AML enrolled in the trial, including age, pathogenic gene mutations, and bone marrow blast percentage. (**D**) Representative case illustrating bone marrow blast percentage before and after XY0206 treatment. Prior regimens included azacitidine (AZA), homoharringtonine plus cytarabine (HA), daunorubicin plus cytarabine (DA), sorafenib (Sora), venetoclax (Ven), homoharringtonine plus cytarabine plus daunorubicin (HAD), intermediate-dose cytarabine (IDAC), and decitabine (DAC). Patients 13 and 14 proceeded to HSCT. (**E**) Dynamic changes in bone marrow (BM) and peripheral blood (PB) blast percentages and hematologic parameters, including hemoglobin (HGB), platelet count (PLT), WBC count, and RBC count, during XY0206 treatment in 3 representative patients. Measurements were collected at baseline and multiple time points after treatment initiation.

**Table 3:**
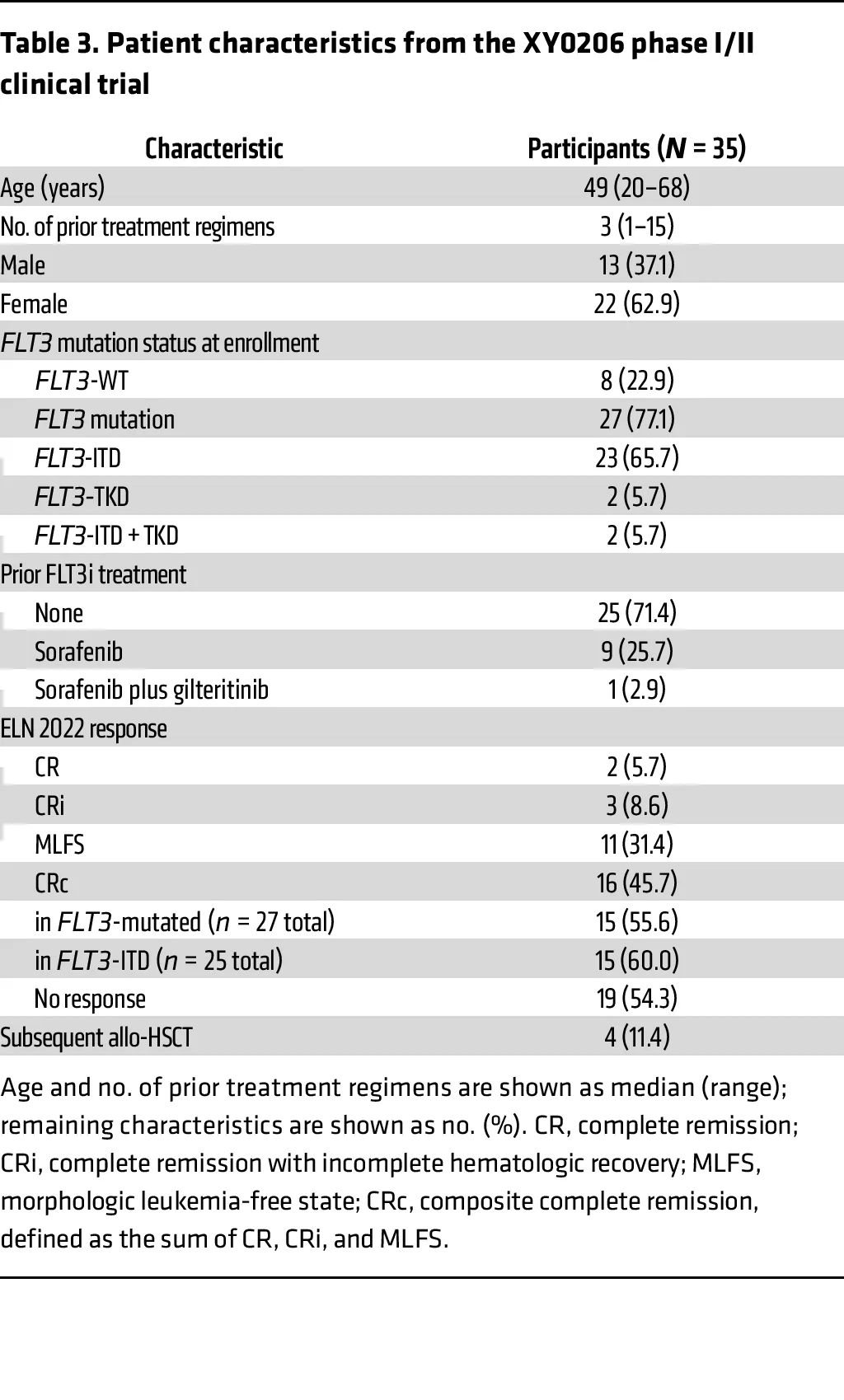
Patient characteristics from the XY0206 phase I/II clinical trial

**Table 5:**
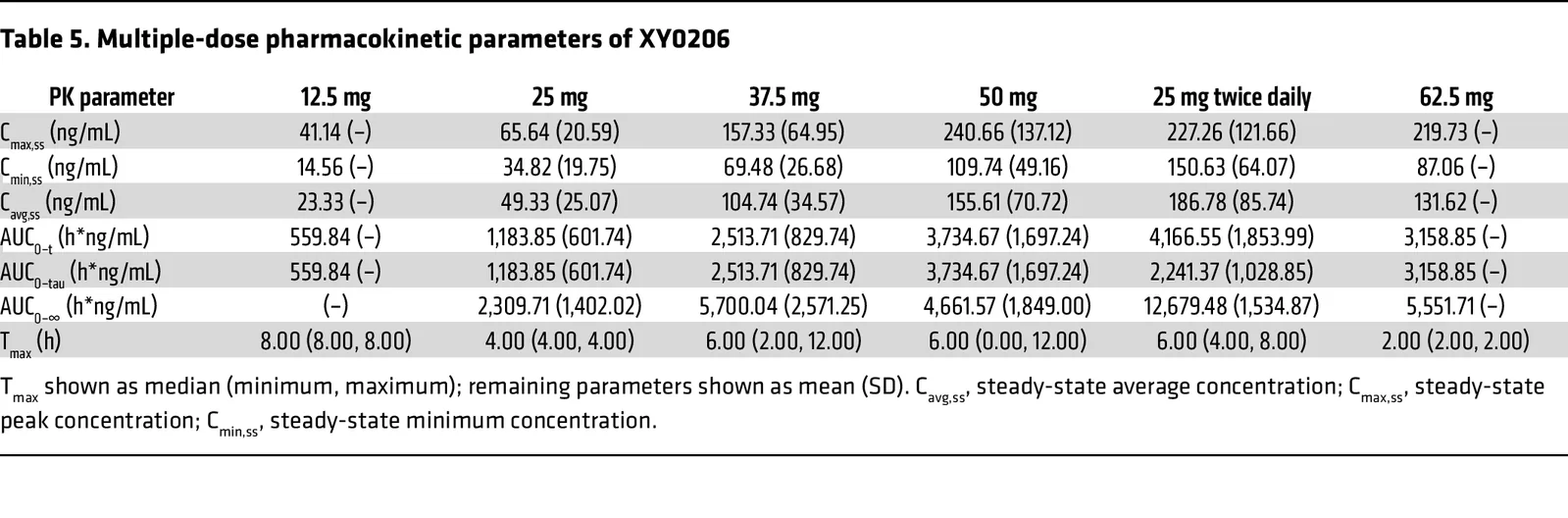
Multiple-dose pharmacokinetic parameters of XY0206

**Table 2:**
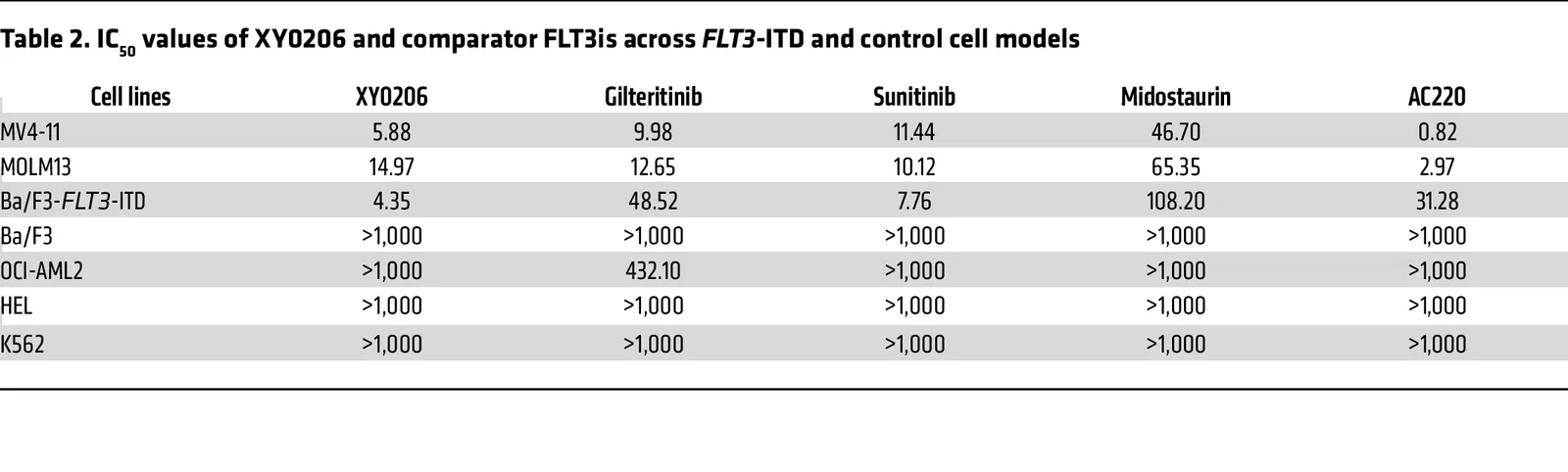
IC_50_ values of XY0206 and comparator FLT3is across *FLT3*-ITD and control cell models

**Table 1:**
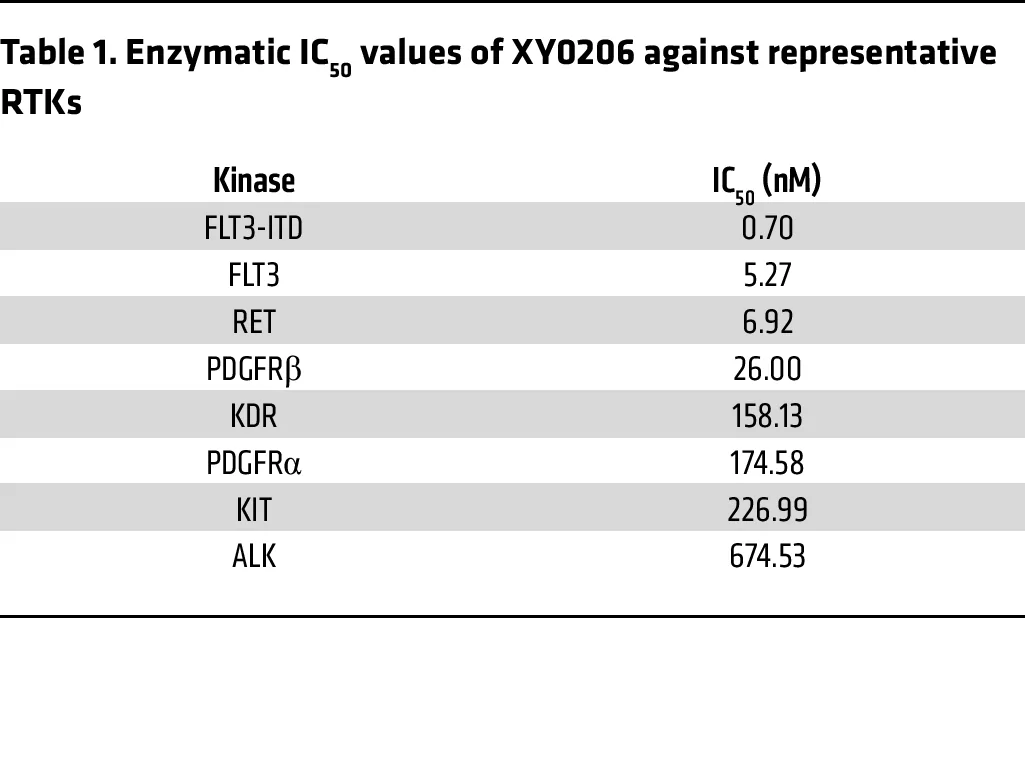
Enzymatic IC_50_ values of XY0206 against representative RTKs

**Table 4:**
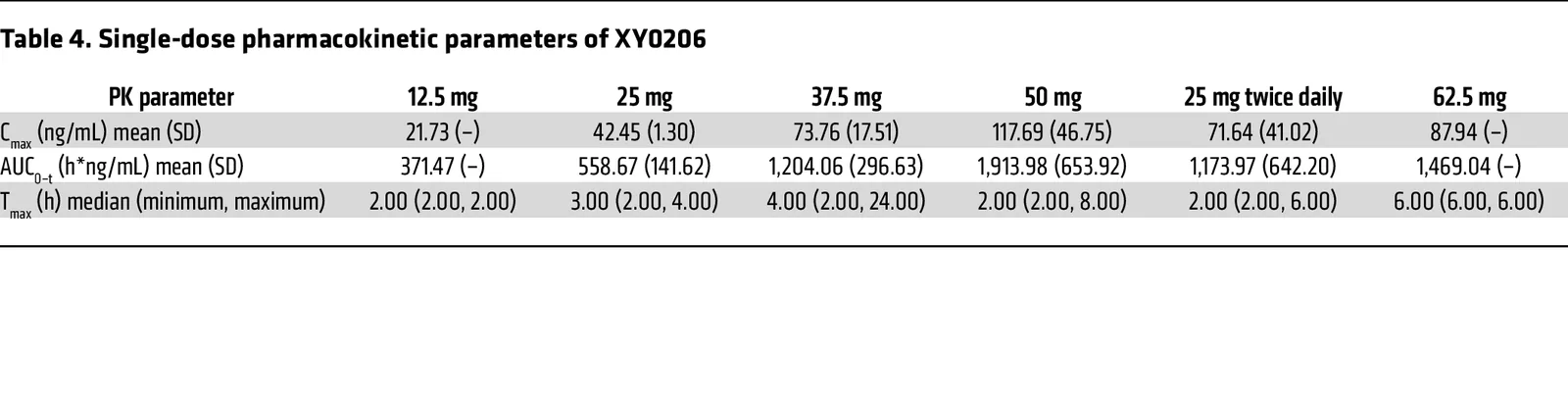
Single-dose pharmacokinetic parameters of XY0206

**Table 6:**
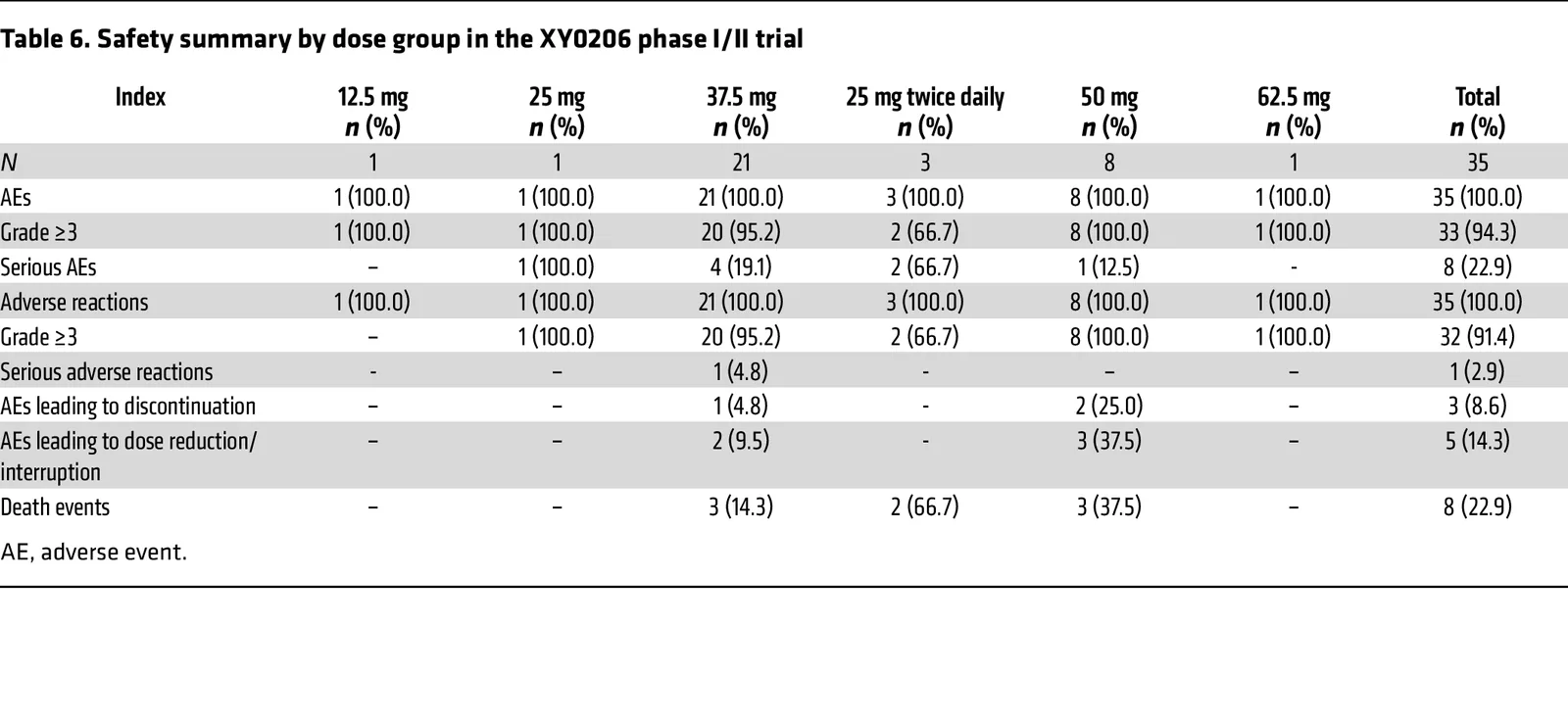
Safety summary by dose group in the XY0206 phase I/II trial

## Version 1 09/01/2026

In-Press Preview

## Version 2 10/15/2026

Electronic publication
