# Supplementary material for: XY0206 targets FLT3-dependent resistance states in acute myeloid leukemia
Source: J Clin Invest. 2026 Sep 1;136(20):e204701. doi: 10.1172/JCI204701 (PMC13668629; doi:10.1172/JCI204701)
Supplement: Supplementary file 1 [file jci-136-204701-s198.pdf]

Supplemental Figures

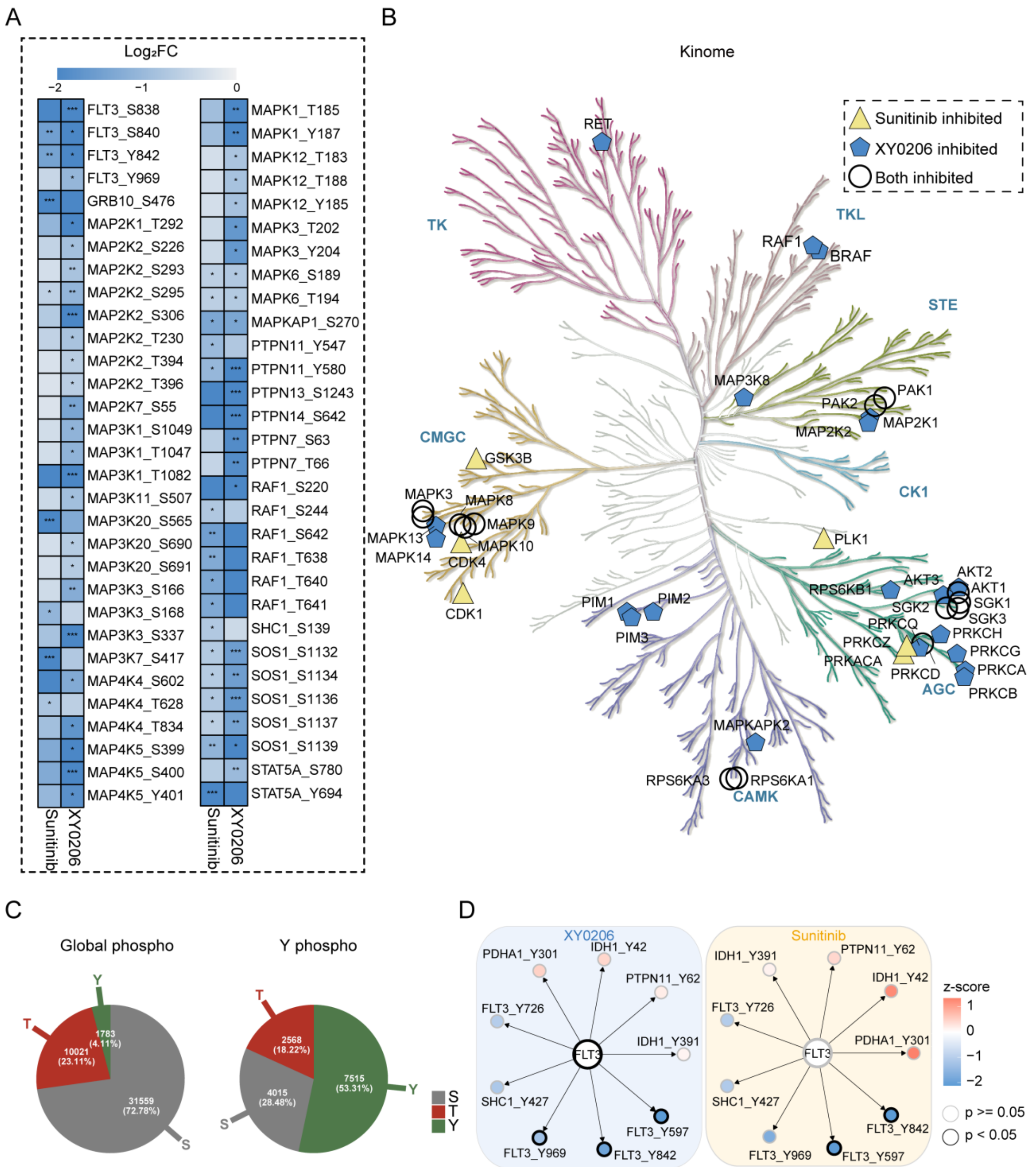

Supplemental Figure 1. Comparative analysis of FLT3 signaling modulation by XY0206 and sunitinib. (A) Heatmap depicting differentially regulated phosphosites identified by global

phosphoproteomic profiling after treatment with XY0206 or sunitinib. Phosphosites showing decreased phosphorylation relative to vehicle control are indicated in blue. Statistical significance is indicated as *$P < 0.05$, **$P < 0.01$, and ***$P < 0.001$. (B) Kinase activity tree inferred from differentially regulated phosphosites in the global phosphoproteomic dataset after XY0206 or sunitinib treatment. Kinase activity was inferred using KSEA and RoKAI. Differentially regulated kinases were defined as overlapping kinases identified by both KSEA and RoKAI, with $P < 0.05$ and a minimum substrate count of 2. (C) Pie charts compare the proportional distribution and absolute number of identified serine (S), threonine (T), and tyrosine (Y) phosphorylation sites in the global phosphoproteomic dataset and the tyrosine enriched phosphoproteomic dataset. (D) FLT3 phosphorylation changes inferred from the tyrosine enriched phosphoproteomic dataset after treatment with XY0206 or sunitinib. Blue intensity indicates the degree of phosphorylation inhibition.

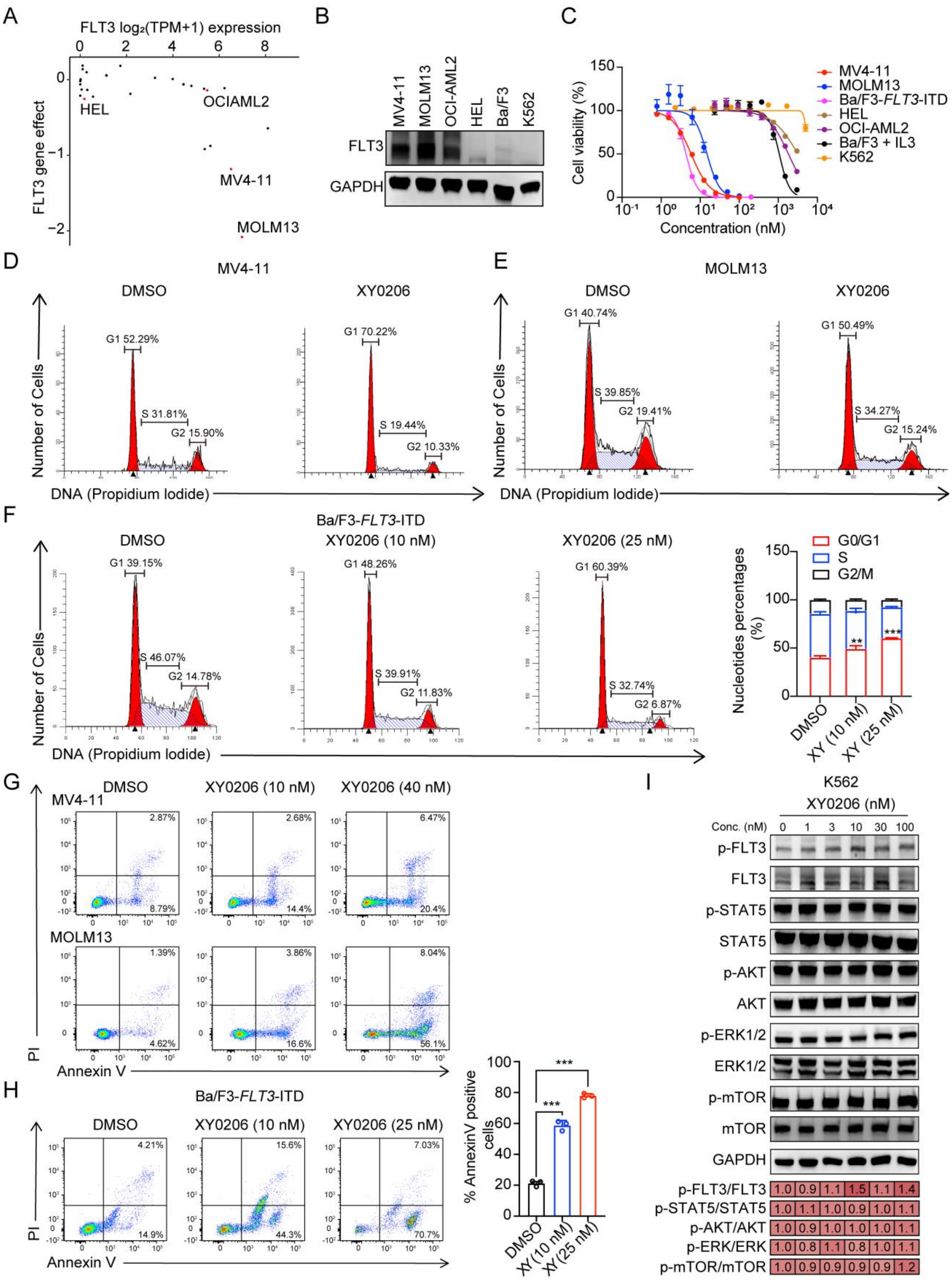
A
FLT3 log₂(TPM+1) expression
FLT3 gene effect
HEL
OCIAML2
MV4-11
MOLM13
B
MV4-11
MOLM13
OCI-AML2
HEL
Ba/F3
K562
FLT3
GAPDH
C
Cell viability (%)
Concentration (nM)
MV4-11
MOLM13
Ba/F3-FLT3-ITD
HEL
OCI-AML2
Ba/F3 + IL3
K562
D
MV4-11
DMSO
XY0206
G1 52.29%
S 31.81%
G2 15.90%
G1 70.22%
S 19.44%
G2 10.33%
Number of Cells
DNA (Propidium Iodide)
E
MOLM13
DMSO
XY0206
G1 40.74%
S 39.85%
G2 19.41%
G1 50.49%
S 34.27%
G2 15.24%
Number of Cells
DNA (Propidium Iodide)
F
Ba/F3-FLT3-ITD
DMSO
XY0206 (10 nM)
XY0206 (25 nM)
G1 39.15%
S 46.07%
G2 14.78%
G1 48.26%
S 39.91%
G2 11.83%
G1 60.39%
S 32.74%
G2 6.87%
Number of Cells
DNA (Propidium Iodide)
G0/G1
S
G2/M
Nucleotides percentages (%)
DMSO
XY (10 nM)
XY (25 nM)
G
DMSO
XY0206 (10 nM)
XY0206 (40 nM)
MV4-11
2.87%
8.79%
2.68%
14.4%
6.47%
20.4%
MOLM13
1.39%
4.62%
3.86%
16.6%
8.04%
56.1%
PI
Annexin V
H
Ba/F3-FLT3-ITD
DMSO
XY0206 (10 nM)
XY0206 (25 nM)
4.21%
14.9%
15.6%
44.3%
7.03%
70.7%
PI
Annexin V
% AnnexinV positive cells
DMSO
XY (10 nM)
XY (25 nM)
I
K562
XY0206 (nM)
Conc. (nM) 0 1 3 10 30 100
p-FLT3
FLT3
p-STAT5
STAT5
p-AKT
AKT
p-ERK1/2
ERK1/2
p-mTOR
mTOR
GAPDH
p-FLT3/FLT3 1.0 0.9 1.1 1.5 1.1 1.4
p-STAT5/STAT5 1.0 1.1 1.0 0.9 1.0 1.1
p-AKT/AKT 1.0 0.9 1.0 1.0 1.0 1.1
p-ERK/ERK 1.0 0.8 1.1 0.8 1.0 1.1
p-mTOR/mTOR 1.0 0.9 0.9 0.9 0.9 1.2

Supplemental Figure 2. Anti-leukemic activity of XY0206 in *FLT3*-ITD and control cell models. (A) DepMap transcriptomic analysis showing relative FLT3 mRNA expression levels and FLT3 gene dependency scores across the indicated AML cell lines. (B) Immunoblot analysis of FLT3 protein expression in the indicated leukemia cell lines. (C) Cell viability of *FLT3*-ITD-mutated cells (MV4-11, MOLM13, and Ba/F3-*FLT3*-ITD) and *FLT3*-wild-type or *FLT3*-negative cells (OCI-AML2, HEL, K562, and parental Ba/F3) following 3-day treatment with XY0206. (D and E) Representative cell-cycle profiles of *FLT3*-ITD AML cell lines (MV4-11 and MOLM13) treated with DMSO or XY0206 for 24 hours. Cell-cycle phases were quantified using ModFit LT v3.2. (F) Quantification and representative plots of cell-cycle distribution in Ba/F3-*FLT3*-ITD cells after 24-hour treatment with DMSO or XY0206, analyzed by PI staining and flow cytometry. (G) Representative apoptosis analysis of *FLT3*-ITD AML cells (MV4-11 and MOLM13) after 48-hour treatment with XY0206, assessed by Annexin V/PI staining and flow cytometry. (H) Quantification and representative plots of apoptosis in Ba/F3-*FLT3*-ITD cells following 72-hour XY0206 treatment, assessed by Annexin V/PI staining and flow cytometry. (I) Immunoblot analysis of total and phosphorylated FLT3, STAT5, AKT, ERK1/2, and mTOR in FLT3-independent K562 cells treated with XY0206 at the indicated doses for 3 hours. Densitometric ratios of phosphorylated to total protein were quantified using ImageJ and expressed as fold changes relative to vehicle control. Data are shown as mean ± SD from 3 independent biological experiments for C, F, and H. Statistical analyses were performed using 1-way ANOVA followed by Dunnett's multiple-comparison test for F and H. $^{**}P < 0.01$, $^{***}P < 0.001$. All immunoblots were repeated independently at least twice with similar results, and representative blots are shown.

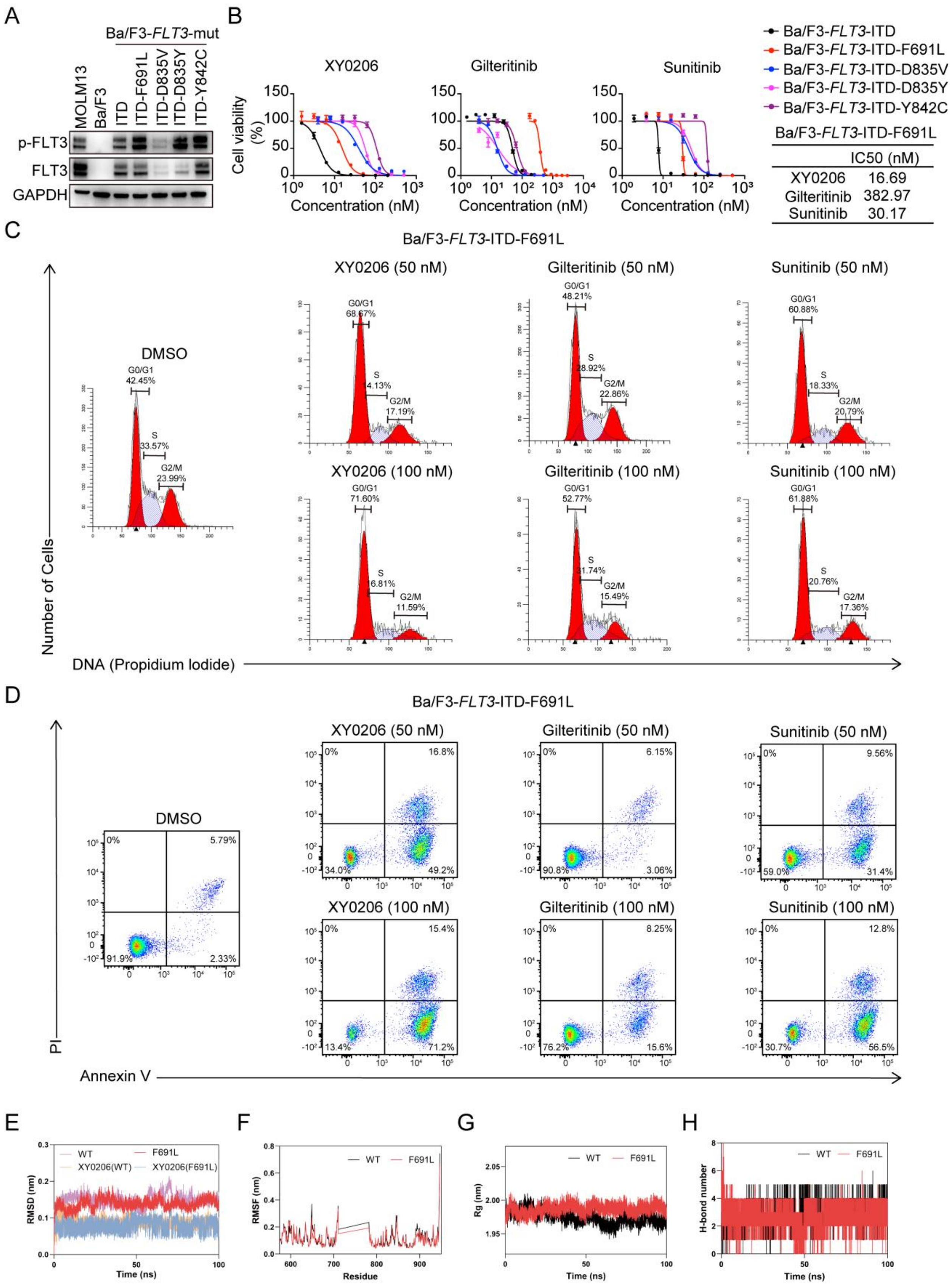
A
Ba/F3-FLT3-mut
MOLM13
Ba/F3
ITD
ITD-F691L
ITD-D835V
ITD-D835Y
ITD-Y842C
p-FLT3
FLT3
GAPDH
B
XY0206
Gilteritinib
Sunitinib
Cell viability (%)
Concentration (nM)
Ba/F3-FLT3-ITD
Ba/F3-FLT3-ITD-F691L
Ba/F3-FLT3-ITD-D835V
Ba/F3-FLT3-ITD-D835Y
Ba/F3-FLT3-ITD-Y842C
Ba/F3-FLT3-ITD-F691L
IC50 (nM)
XY0206 16.69
Gilteritinib 382.97
Sunitinib 30.17
C
Ba/F3-FLT3-ITD-F691L
DMSO
G0/G1 42.45%
S 33.57%
G2/M 23.99%
XY0206 (50 nM)
G0/G1 68.67%
S 4.13%
G2/M 17.19%
Gilteritinib (50 nM)
G0/G1 48.21%
S 28.92%
G2/M 22.86%
Sunitinib (50 nM)
G0/G1 60.88%
S 18.33%
G2/M 20.79%
XY0206 (100 nM)
G0/G1 71.60%
S 16.81%
G2/M 11.59%
Gilteritinib (100 nM)
G0/G1 52.77%
S 31.74%
G2/M 15.49%
Sunitinib (100 nM)
G0/G1 61.88%
S 20.76%
G2/M 17.36%
Number of Cells
DNA (Propidium Iodide)
D
Ba/F3-FLT3-ITD-F691L
DMSO
0% 5.79% 91.9% 2.33%
XY0206 (50 nM)
0% 16.8% 34.0% 49.2%
Gilteritinib (50 nM)
0% 6.15% 90.8% 3.06%
Sunitinib (50 nM)
0% 9.56% 59.0% 31.4%
XY0206 (100 nM)
0% 15.4% 13.4% 71.2%
Gilteritinib (100 nM)
0% 8.25% 76.2% 15.6%
Sunitinib (100 nM)
0% 12.8% 30.7% 56.5%
PI
Annexin V
E
WT
F691L
XY0206(WT)
XY0206(F691L)
RMSD (nm)
Time (ns)
F
WT
F691L
RMSF (nm)
Residue
G
WT
F691L
Rg (nm)
Time (ns)
H
WT
F691L
H-bond number
Time (ns)

Supplemental Figure 3. Activity of XY0206 against *FLT3*-ITD cells harboring the F691L gatekeeper mutation. (A) Immunoblot analysis of total FLT3 and phosphorylated FLT3 (p-FLT3) in Ba/F3 cells expressing *FLT3*-ITD alone or in combination with representative tyrosine kinase domain mutations, including F691L, D835V, D835Y, and Y842C. MOLM13 cells were included as a positive control for endogenous *FLT3*-ITD expression. (B) Cell viability of Ba/F3-*FLT3*-ITD cells with or without TKD mutations following 72-hour treatment with XY0206, gilteritinib or sunitinib, assessed using the CCK-8 assay. IC_{50} values were calculated using GraphPad Prism. (C) Representative cell cycle profiles of Ba/F3-*FLT3*-ITD-F691L cells following 24-hour treatment with XY0206, gilteritinib or sunitinib, analyzed by PI staining and flow cytometry. Cell cycle distribution was determined using ModFit LT software (version 3.2). (D) Representative flow cytometric analysis of apoptosis in Ba/F3-*FLT3*-ITD-F691L cells after 48-hour treatment with XY0206, gilteritinib or sunitinib, assessed by Annexin V/PI staining. (E) Root mean square deviation (RMSD) analysis of FLT3-WT and FLT3-F691L in complex with XY0206 during a 100-ns molecular dynamics simulation. (F) Root mean square fluctuation (RMSF) analysis of FLT3-WT and FLT3-F691L in complex with XY0206. Residues 692–781 are absent from the crystal structure. (G) Radius of gyration (Rg) analysis of FLT3-WT and FLT3-F691L in complex with XY0206 during molecular dynamics simulation. (H) Hydrogen bond analysis between XY0206 and FLT3-F691L during molecular dynamics simulation. The number of hydrogen bonds remained between 2 and 4 throughout the simulation, indicating sustained ligand–protein interactions. Data are shown as mean ± SD from 3 independent biological experiments for B. All immunoblots were repeated independently at least twice with similar results, and representative blots are shown.

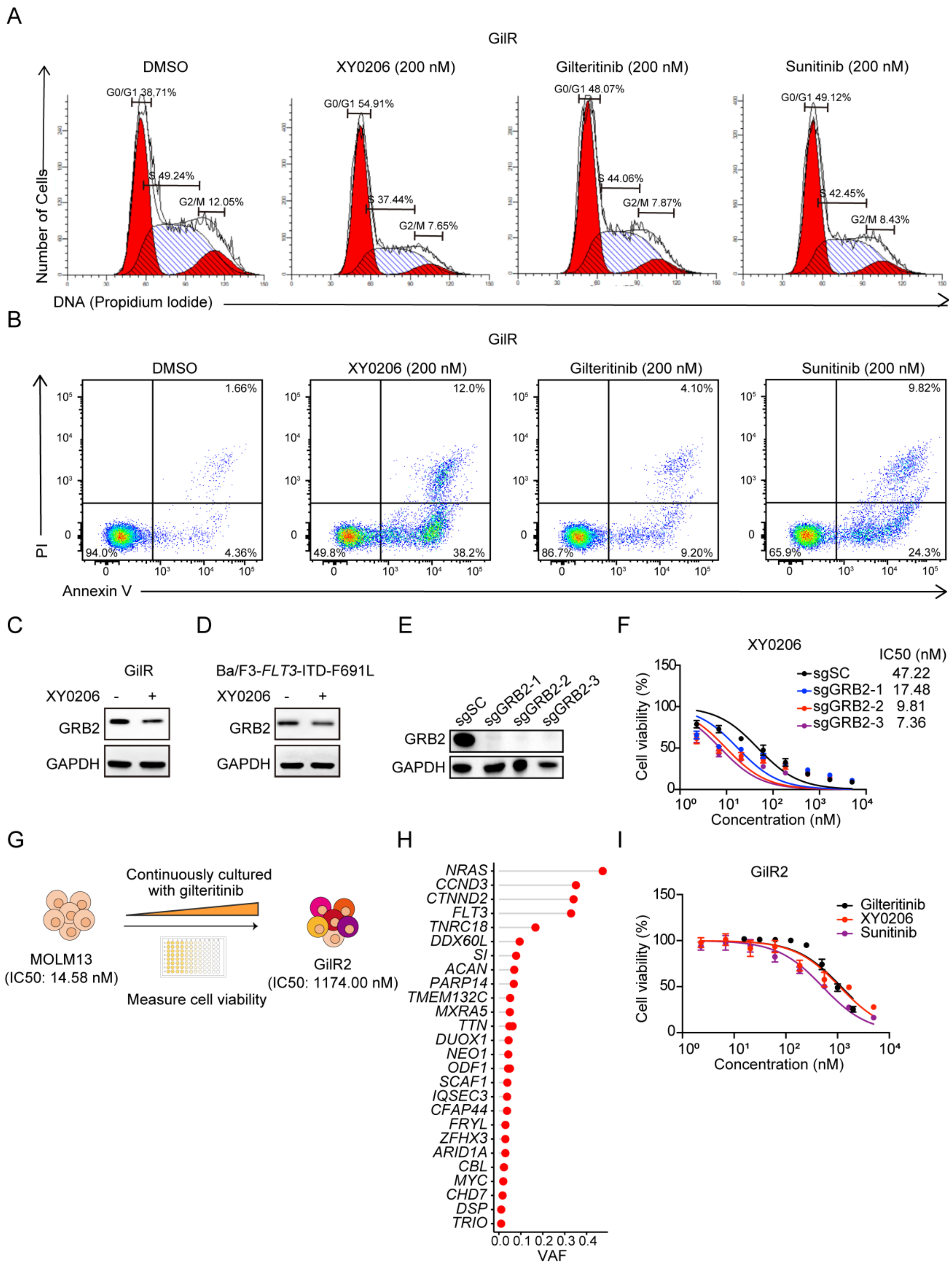

A
GilR
DMSO
XY0206 (200 nM)
Gilteritinib (200 nM)
Sunitinib (200 nM)
G0/G1 38.71%
S 49.24%
G2/M 12.05%
G0/G1 54.91%
S 37.44%
G2/M 7.65%
G0/G1 48.07%
S 44.06%
G2/M 7.87%
G0/G1 49.12%
S 42.45%
G2/M 8.43%
Number of Cells
DNA (Propidium Iodide)
B
GilR
DMSO
XY0206 (200 nM)
Gilteritinib (200 nM)
Sunitinib (200 nM)
1.66%
94.0%
4.36%
12.0%
49.8%
38.2%
4.10%
86.7%
9.20%
9.82%
65.9%
24.3%
PI
Annexin V
C
GilR
XY0206 - +
GRB2
GAPDH
D
Ba/F3-FLT3-ITD-F691L
XY0206 - +
GRB2
GAPDH
E
sgSC sgGRB2-1 sgGRB2-2 sgGRB2-3
GRB2
GAPDH
F
XY0206
IC50 (nM)
sgSC 47.22
sgGRB2-1 17.48
sgGRB2-2 9.81
sgGRB2-3 7.36
Cell viability (%)
Concentration (nM)
G
Continuously cultured with gilteritinib
MOLM13 (IC50: 14.58 nM)
Measure cell viability
GilR2 (IC50: 1174.00 nM)
H
NRAS
CCND3
CTNND2
FLT3
TNRC18
DDX60L
SI
ACAN
PARP14
TMEM132C
MXRA5
TTN
DUOX1
NEO1
ODF1
SCAF1
IQSEC3
CFAP44
FRYL
ZFHX3
ARID1A
CBL
MYC
CHD7
DSP
TRIO
VAF
I
GilR2
Gilteritinib
XY0206
Sunitinib
Cell viability (%)
Concentration (nM)

73

Supplemental Figure 4. Characterization of acquired gilteritinib resistance models and GRB2-associated modulation of XY0206 response. (A) Representative cell cycle distribution of gilteritinib-tolerant GilR cells following 24-hour treatment with DMSO, XY0206, gilteritinib or sunitinib. Cell cycle profiles were determined by PI staining and flow cytometry and analyzed using ModFit LT software (version 3.2). (B) Representative flow cytometric analysis of apoptosis in GilR cells after 72-hour treatment with DMSO, XY0206, gilteritinib or sunitinib, assessed by Annexin V/PI staining. (C and D) Immunoblot analysis of GRB2 expression in GilR and Ba/F3-*FLT3*-ITD-F691L cells treated with XY0206 at the corresponding IC_{50} concentrations. GilR cells were treated for 24 hours, and Ba/F3-*FLT3*-ITD-F691L cells were treated for 48 hours. (E) Immunoblot analysis of GRB2 knockdown efficiency in GilR cells. (F) Cell viability of GilR cells with or without GRB2 knockdown following 72-hour treatment with XY0206, assessed using the CCK-8 assay. IC_{50} values were calculated using GraphPad Prism. (G) Schematic representation of GilR2 cell generation and determination of gilteritinib IC_{50} values. (H) Whole-exome sequencing analysis of GilR2 cells showing acquired genetic alterations relative to parental MOLM13 cells. VAF, variant allele frequency. (I) Cell viability of GilR2 cells following 72-hour treatment with XY0206, gilteritinib, or sunitinib, assessed using the CCK-8 assay. Data are shown as mean $\pm$ SD from 3 independent biological experiments for F and I. All immunoblots were repeated independently at least twice with similar results, and representative blots are shown.

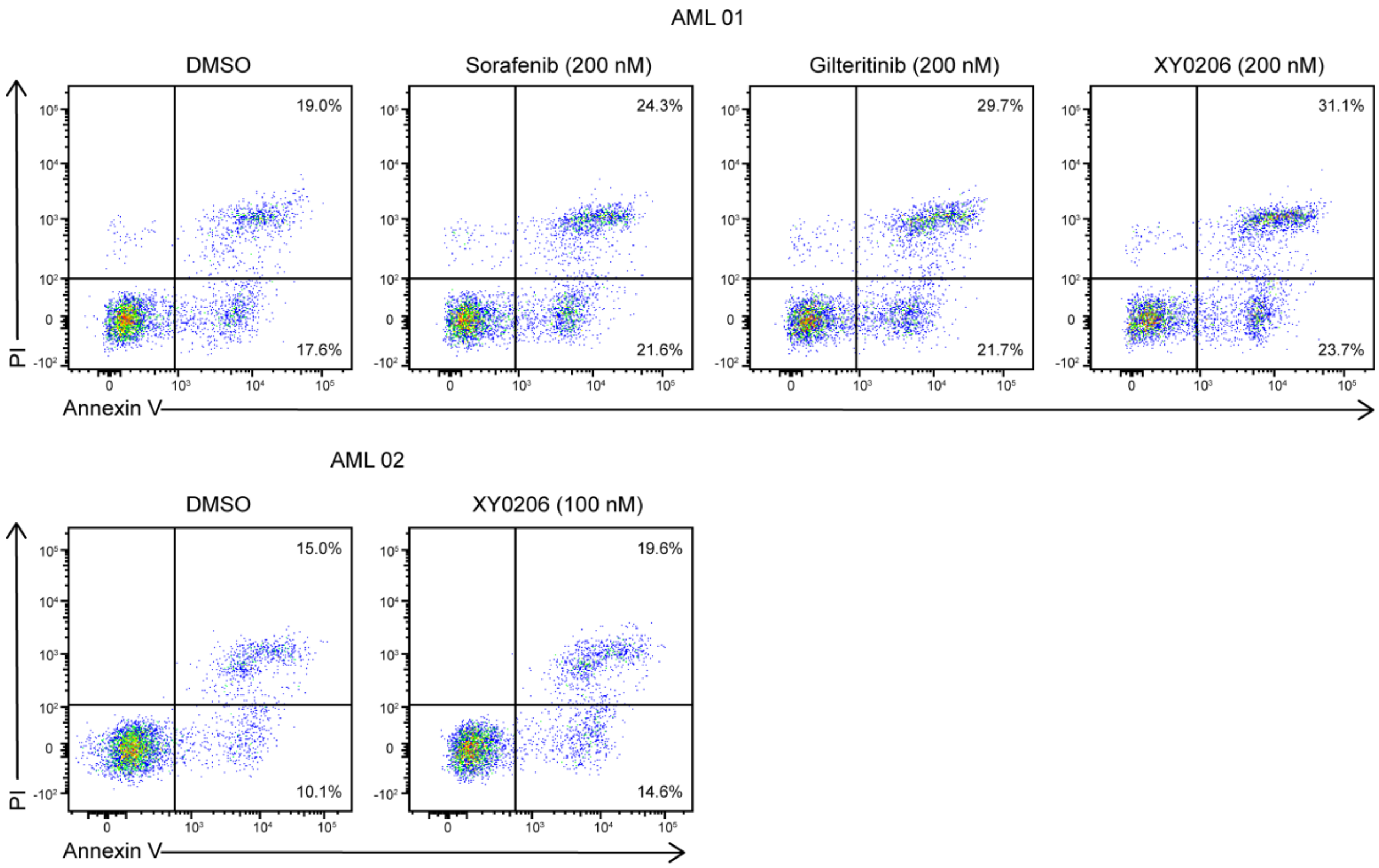

Supplemental Figure 5. Induction of apoptosis in primary AML blasts following XY0206 treatment. Representative flow cytometric analysis of apoptosis in primary AML blasts after 48-hour treatment with DMSO, XY0206, sorafenib, or gilteritinib. Apoptosis was assessed by Annexin V/PI staining.

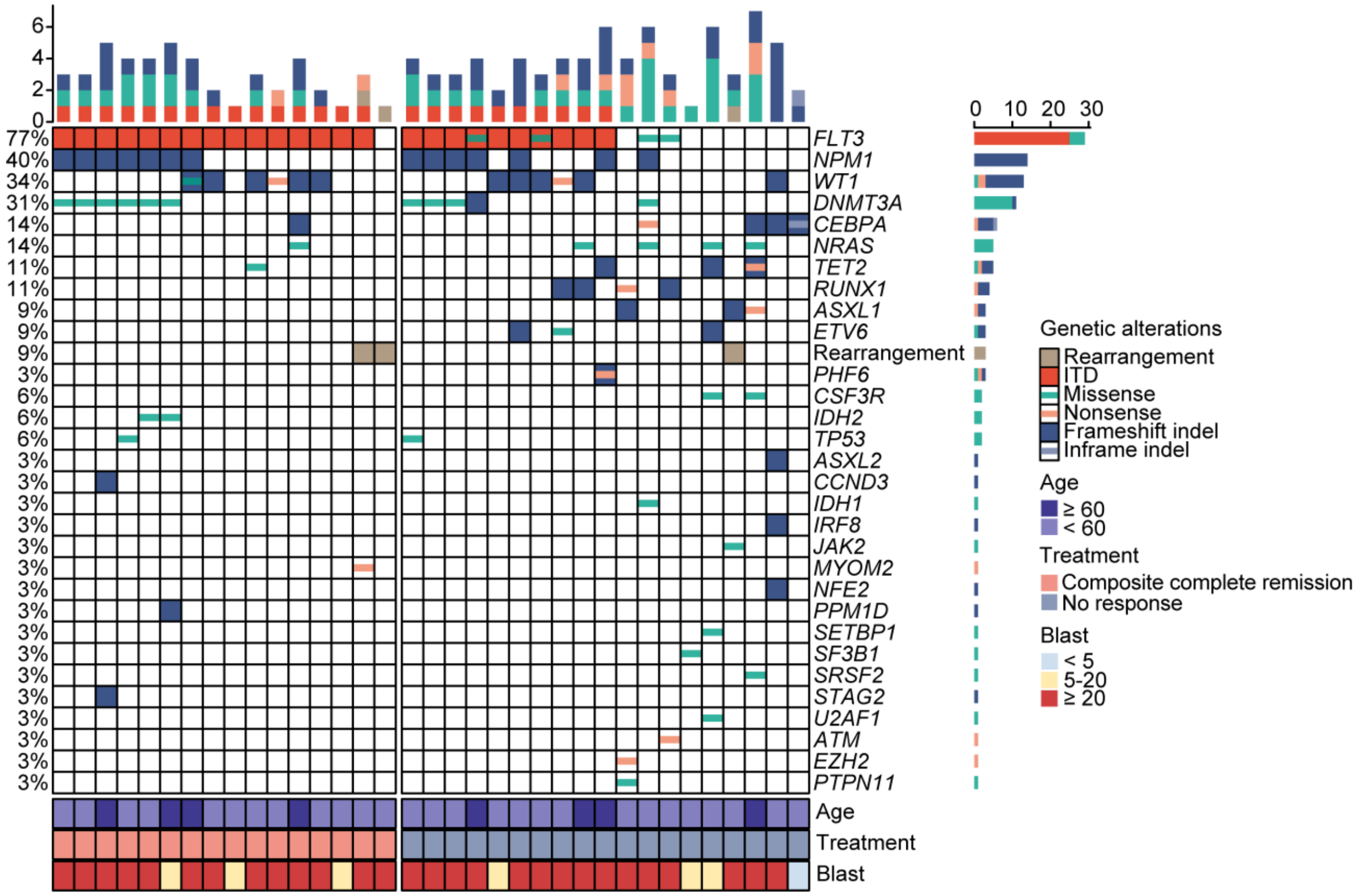

Supplemental Figure 6. Clinical and molecular characteristics of patients with relapsed or refractory AML treated with XY0206. Heatmap summarizing baseline clinical and molecular features of patients with relapsed or refractory AML enrolled in the single-center XY0206 monotherapy trial. Each column represents an individual patient, and each row denotes a specific clinical or genetic characteristic. The main panel shows recurrent genetic and cytogenetic alterations identified in leukemic blasts. The lower annotation tracks show age, treatment response, and baseline blast category, as indicated in the legend. Bar plots summarize alteration counts across patients and genes. Filled boxes indicate the presence of the corresponding feature or alteration.

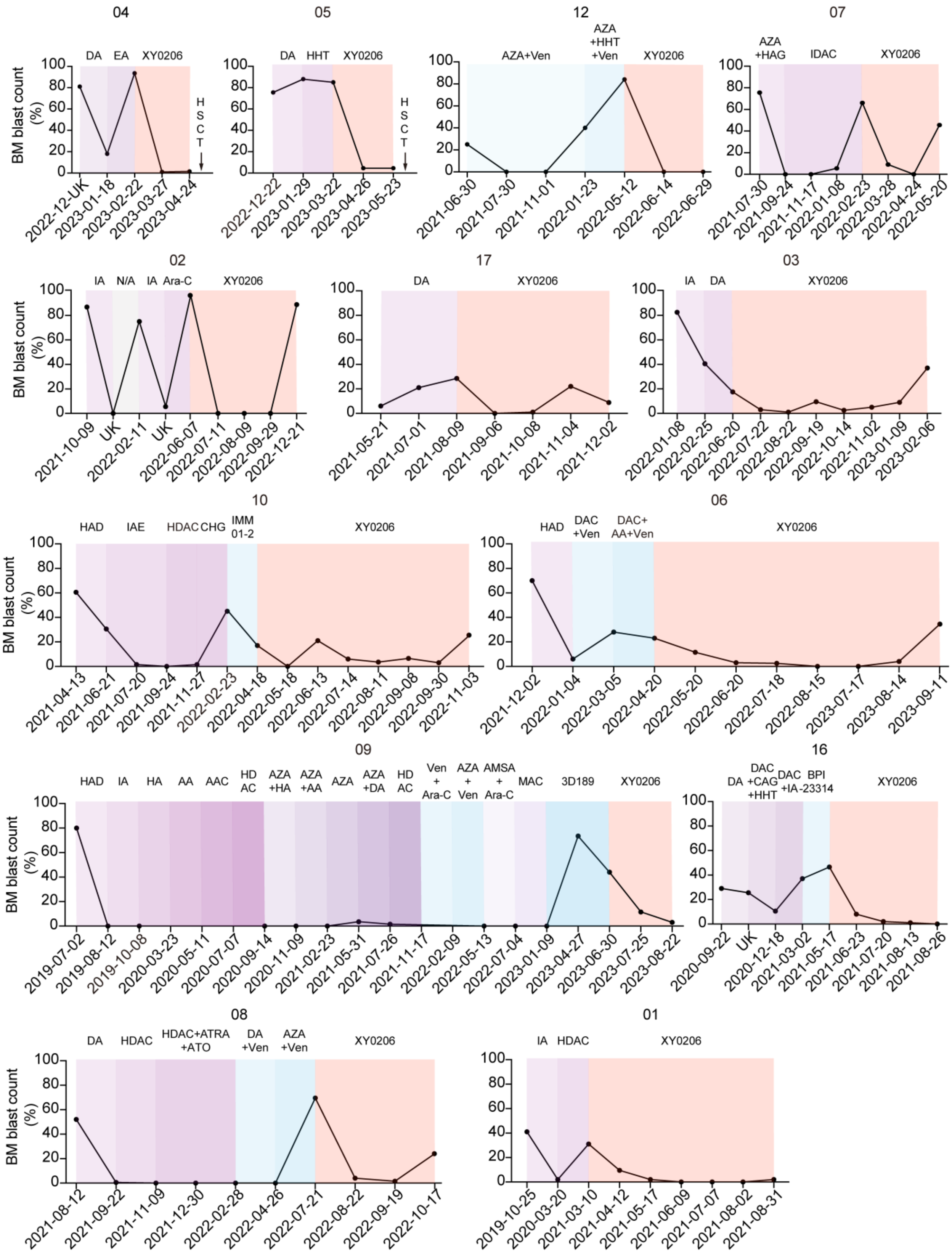
04
DA EA XY0206
HSCT
BM blast count (%)
2022-12-UK 2023-01-18 2023-02-22 2023-03-27 2023-04-24
05
DA HHT XY0206
HSCT
2022-12-22 2023-01-29 2023-03-22 2023-04-26 2023-05-23
12
AZA+Ven
AZA +HHT +Ven
XY0206
2021-06-30 2021-07-30 2021-11-01 2022-01-23 2022-05-12 2022-06-14 2022-06-29
07
AZA +HAG
IDAC
XY0206
2021-07-30 2021-09-24 2021-11-17 2022-01-08 2022-02-23 2022-03-28 2022-04-24 2022-05-20
02
IA N/A IA Ara-C XY0206
2021-10-09 UK 2022-02-11 UK 2022-06-07 2022-07-11 2022-08-09 2022-09-29 2022-12-21
17
DA XY0206
2021-05-21 2021-07-01 2021-08-09 2021-09-06 2021-10-08 2021-11-04 2021-12-02
03
IA DA XY0206
2022-01-08 2022-02-25 2022-06-20 2022-07-22 2022-08-22 2022-09-19 2022-10-14 2022-11-02 2023-01-09 2023-02-06
10
HAD IAE HDAC CHG IMM 01-2 XY0206
2021-04-13 2021-06-21 2021-07-20 2021-09-24 2021-11-27 2022-02-23 2022-04-18 2022-05-18 2022-06-13 2022-07-14 2022-08-11 2022-09-08 2022-09-30 2022-11-03
06
HAD DAC +Ven DAC+ AA+Ven XY0206
2021-12-02 2022-01-04 2022-03-05 2022-04-20 2022-05-20 2022-06-20 2022-07-18 2022-08-15 2023-07-17 2023-08-14 2023-09-11
09
HAD IA HA AA AAC HD AC AZA +HA AZA +AA AZA AZA +DA HD AC Ven + Ara-C AZA + Ven AMSA + Ara-C MAC 3D189 XY0206
2019-07-02 2019-08-12 2019-10-08 2020-03-23 2020-05-11 2020-07-07 2020-09-14 2020-11-09 2021-02-23 2021-05-31 2021-07-26 2021-11-17 2022-02-09 2022-05-13 2022-07-04 2023-01-09 2023-04-27 2023-06-30 2023-07-25 2023-08-22
16
DA DAC +CAG +HHT DAC +IA BPI -23314 XY0206
2020-09-22 UK 2020-12-18 2021-03-02 2021-05-17 2021-06-23 2021-07-20 2021-08-13 2021-08-26
08
DA HDAC HDAC+ATRA +ATO DA +Ven AZA +Ven XY0206
2021-08-12 2021-09-22 2021-11-09 2021-12-30 2022-02-28 2022-04-26 2022-07-21 2022-08-22 2022-09-19 2022-10-17
01
IA HDAC XY0206
2019-10-25 2020-03-20 2021-03-10 2021-04-12 2021-05-17 2021-06-09 2021-07-07 2021-08-02 2021-08-31

Supplemental Figure 7. Longitudinal changes in bone marrow blast percentages in patients achieving CRc during XY0206 treatment. Time course of bone marrow blast percentages in individual patients with relapsed or refractory AML who achieved composite complete remission (CRc) during treatment with XY0206 in the single-center trial. Data points represent serial bone marrow assessments obtained at the indicated time points for each patient. Abbreviations: UK, unknown; DA, daunorubicin plus cytarabine; EA, etoposide plus cytarabine; HHT, homoharringtonine; AZA, azacitidine; IA, idarubicin plus cytarabine; Ara-C, cytarabine; HAD, homoharringtonine plus cytarabine and daunorubicin; IAE, idarubicin plus cytarabine and etoposide; HDAC, high-dose cytarabine; CHG, cytarabine plus homoharringtonine and G-CSF; DAC, decitabine; Ven, venetoclax; AA, aclarubicin plus cytarabine; ATRA, all-trans retinoic acid; ATO, arsenic trioxide; HAG, homoharringtonine plus cytarabine and G-CSF; IDAC, intermediate-dose cytarabine; CAG, aclarubicin plus cytarabine and G-CSF; HA, homoharringtonine plus cytarabine; MAC, mitoxantrone plus cytarabine and cyclophosphamide; AMSA, amsacrine; AAC, aclarubicin plus cytarabine and cladribine. Patients 04 and 05 proceeded to hematopoietic stem cell transplantation (HSCT).

Supplemental Tables

Supplemental Table 1. Enzymatic activity of XY0206 against a 217-kinase panel at 100 nM.

Supplemental Table 2. Differentially expressed proteins identified by quantitative proteomic profiling of *FLT3*-ITD AML cells treated with sunitinib or XY0206.

Supplemental Table 3. Global phosphoproteomic dataset of *FLT3*-ITD AML cells following treatment with sunitinib or XY0206.

Supplemental Table 4. Tyrosine-phosphoproteomic dataset of *FLT3*-ITD AML cells following treatment with sunitinib or XY0206.

Supplemental Table 5. Whole exome sequencing identified genetic alterations in gilteritinib-resistant AML cell models.

Supplemental Table 6. Clinical and molecular characteristics of primary AML samples used for *ex vivo* evaluation of XY0206 activity.

Supplemental Table 7. Clinical and disease characteristics of patients with R/R AML enrolled in the Phase I/II trial of XY0206 monotherapy.

Supplemental Table 8. Incidence of treatment-emergent adverse events in patients with R/R AML treated with XY0206.
