# Supplementary material for: XY0206 targets FLT3-dependent resistance states in acute myeloid leukemia
Source: J Clin Invest. 2026 Sep 1;136(20):e204701. doi: 10.1172/JCI204701 (PMC13668629; doi:10.1172/JCI204701)

Full unedited blot for Figure 1I

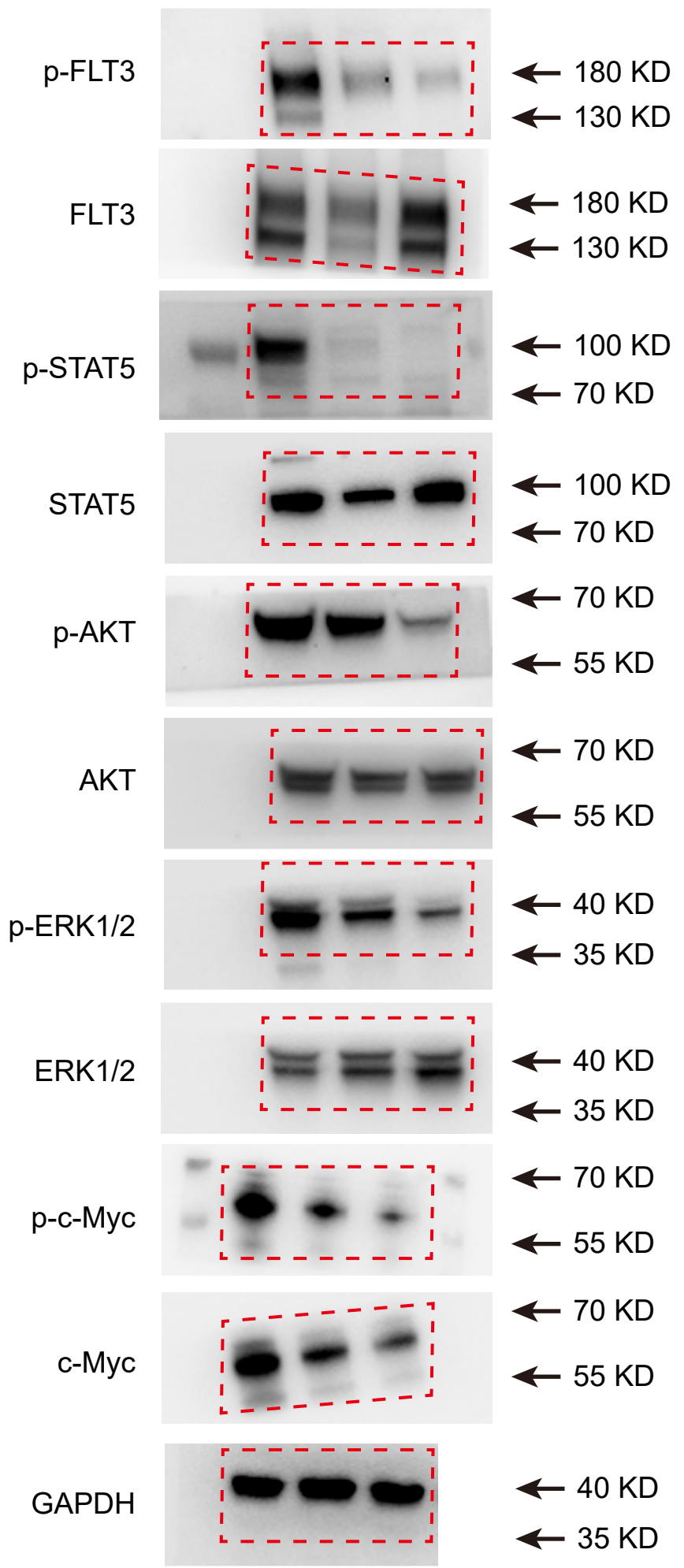

Full unedited blot for Figure 2C

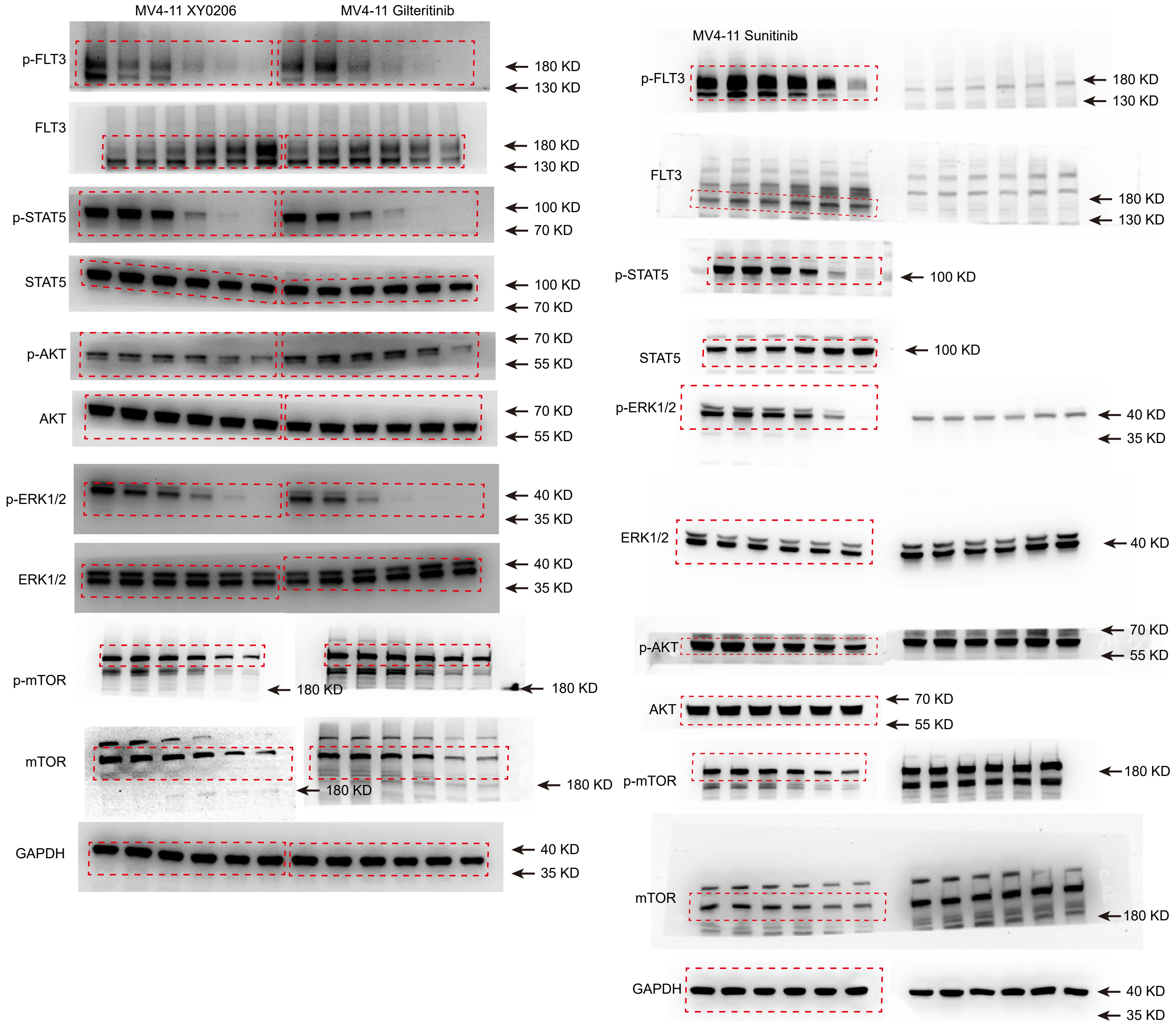

Full unedited blot for Figure 2C

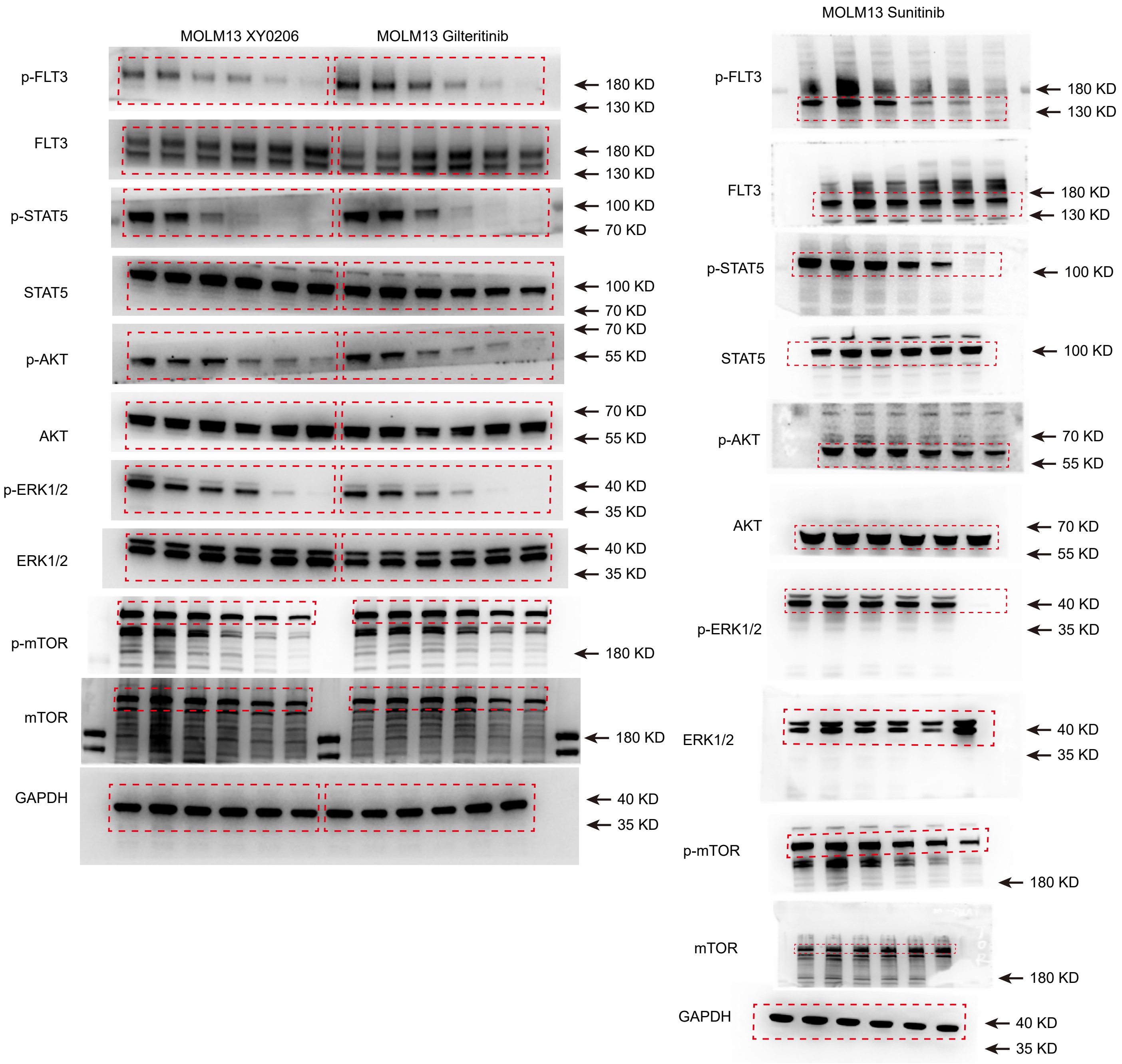

Full unedited blot for Figure 2D

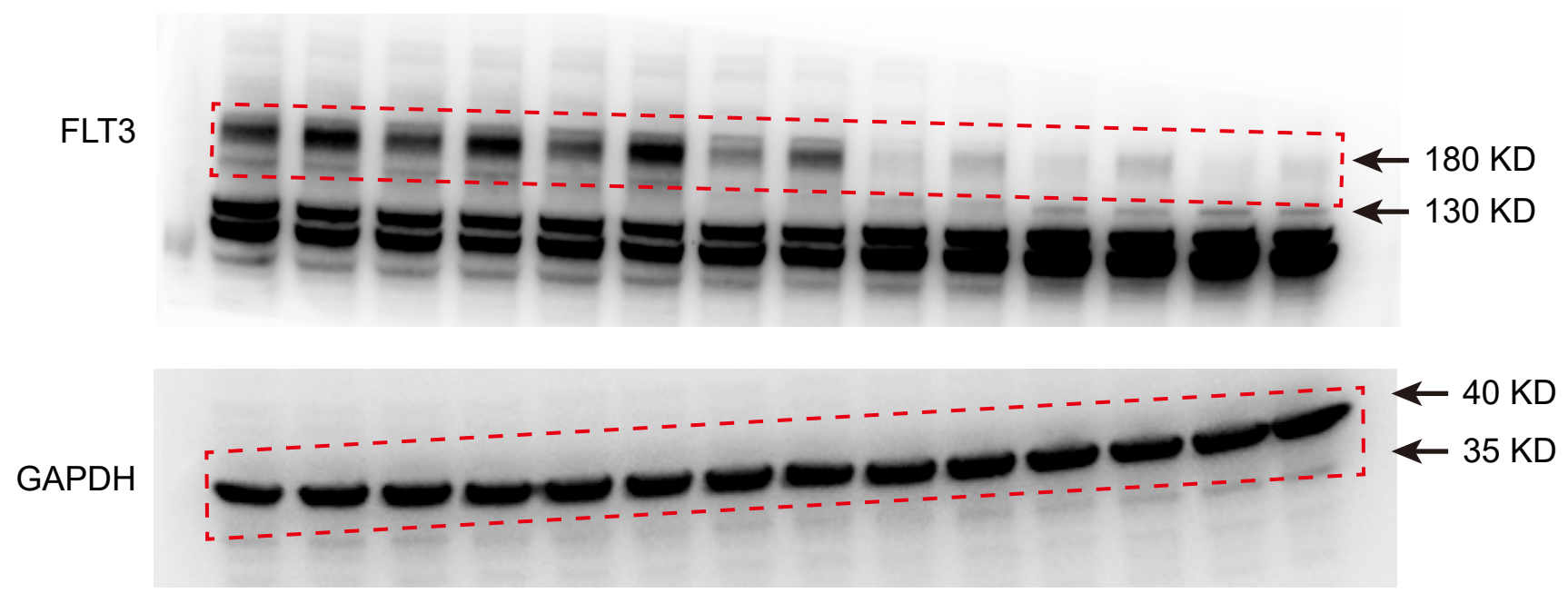

Full unedited blot for Figure 2E

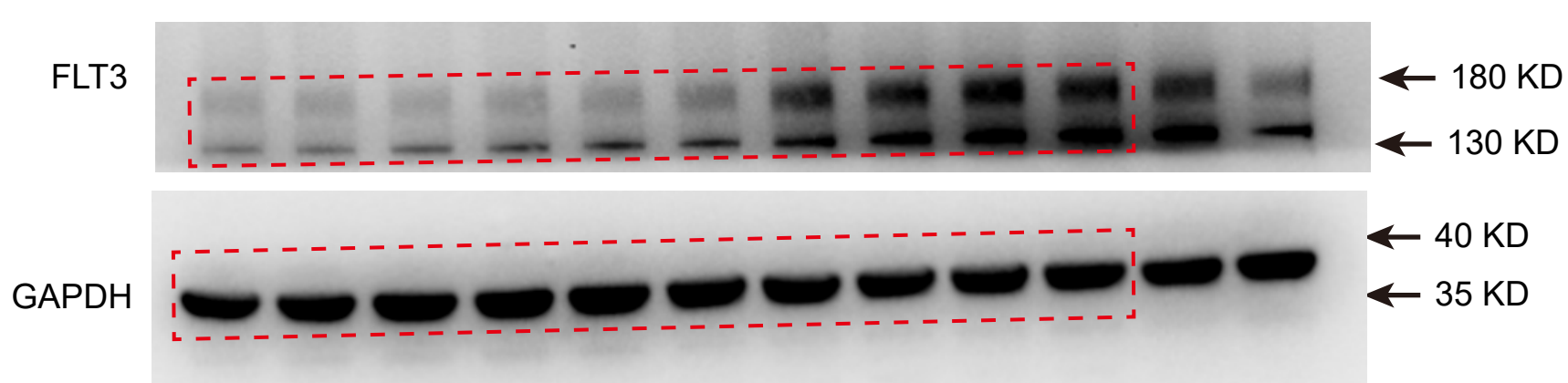

Full unedited blot for Figure 3D

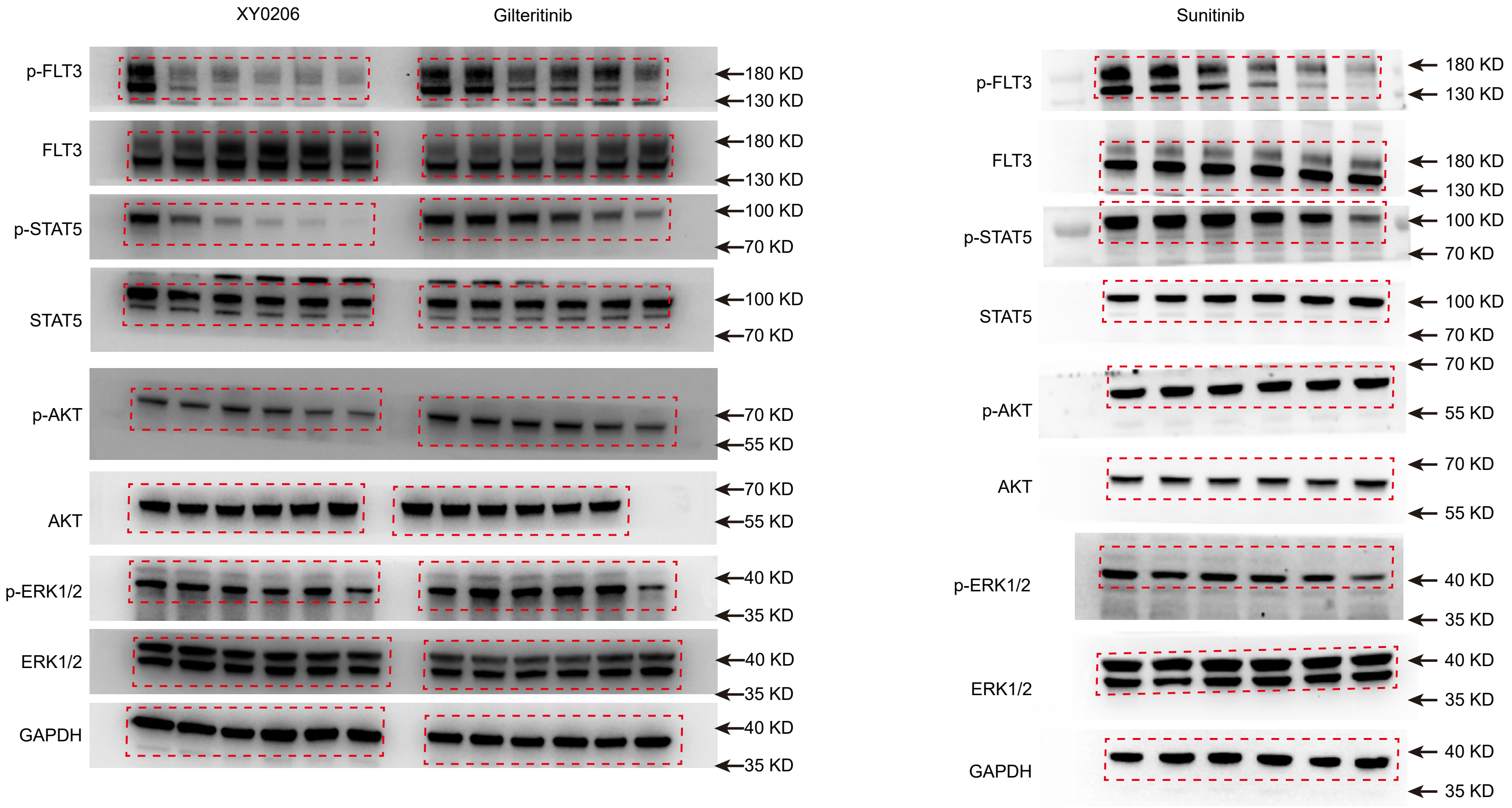

Full unedited blot for Figure 3F

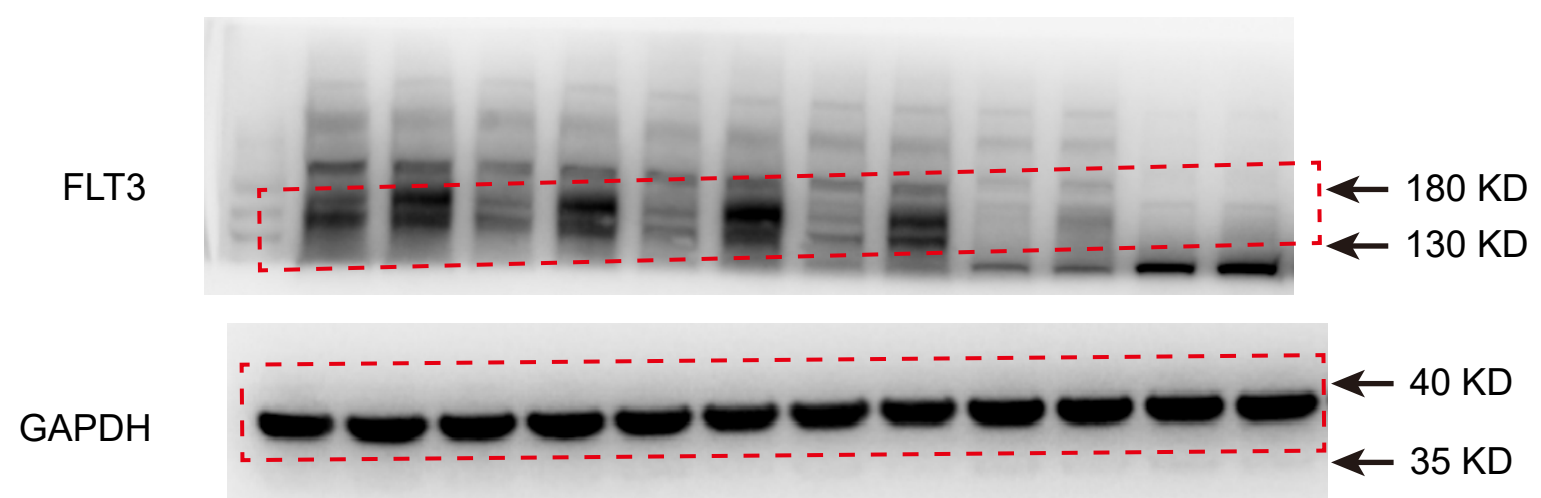

Full unedited blot for Figure 3G

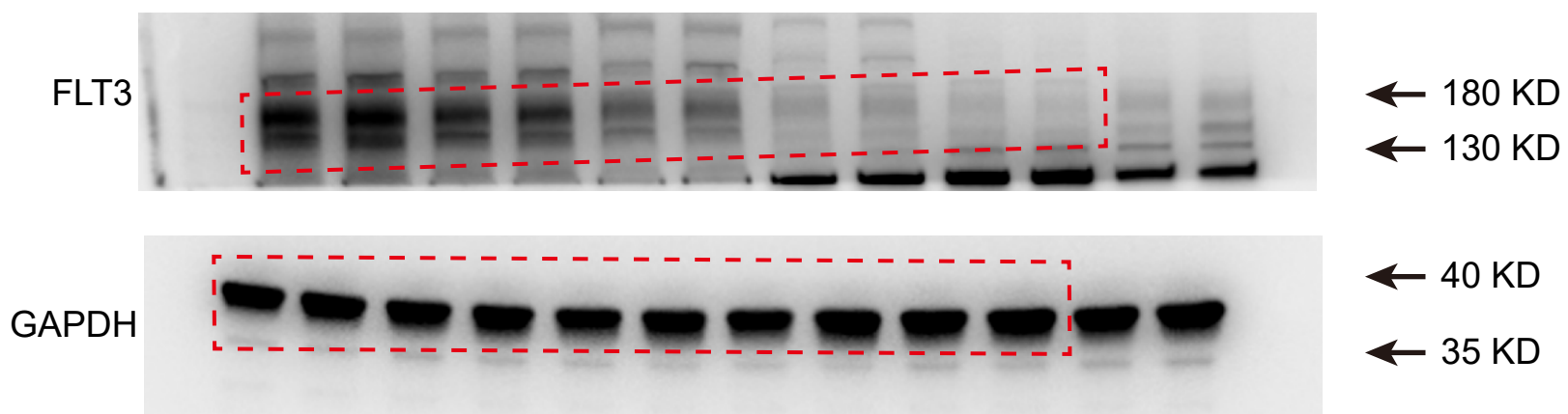

Full unedited blot for Figure 3H

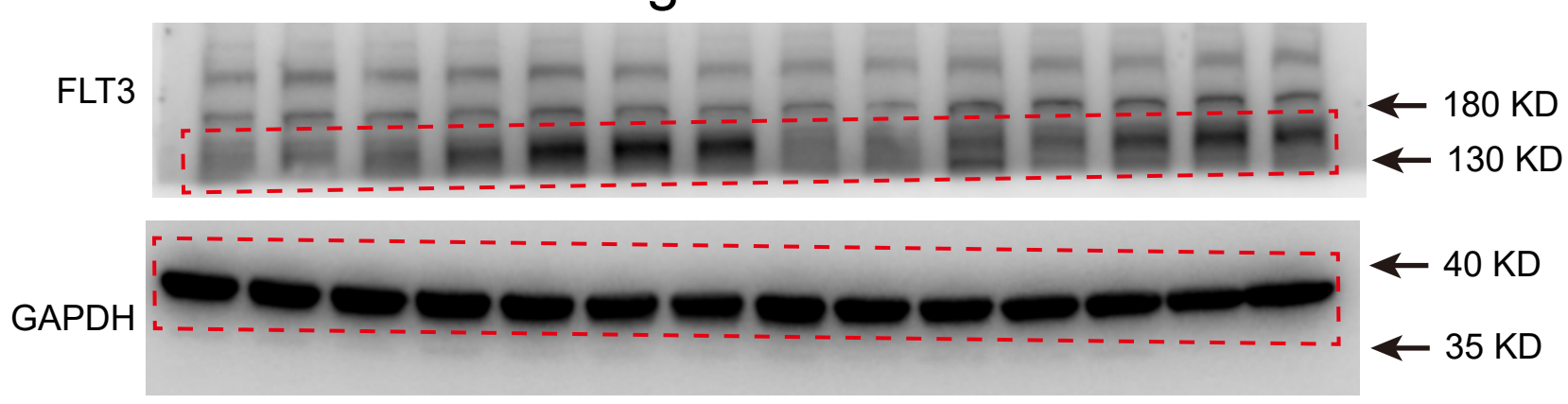

Full unedited blot for Figure 4C

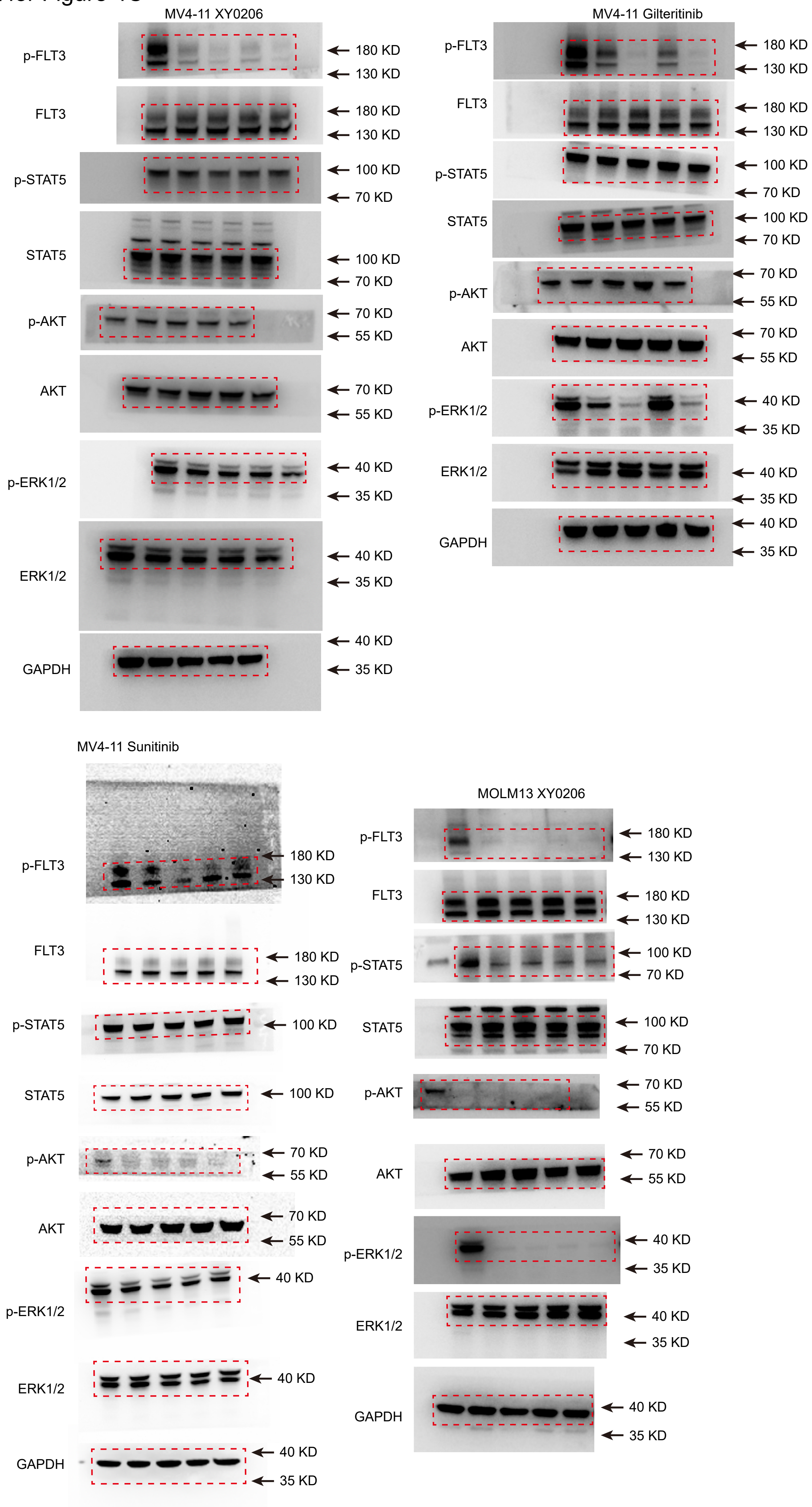

Full unedited blot for Figure 4C

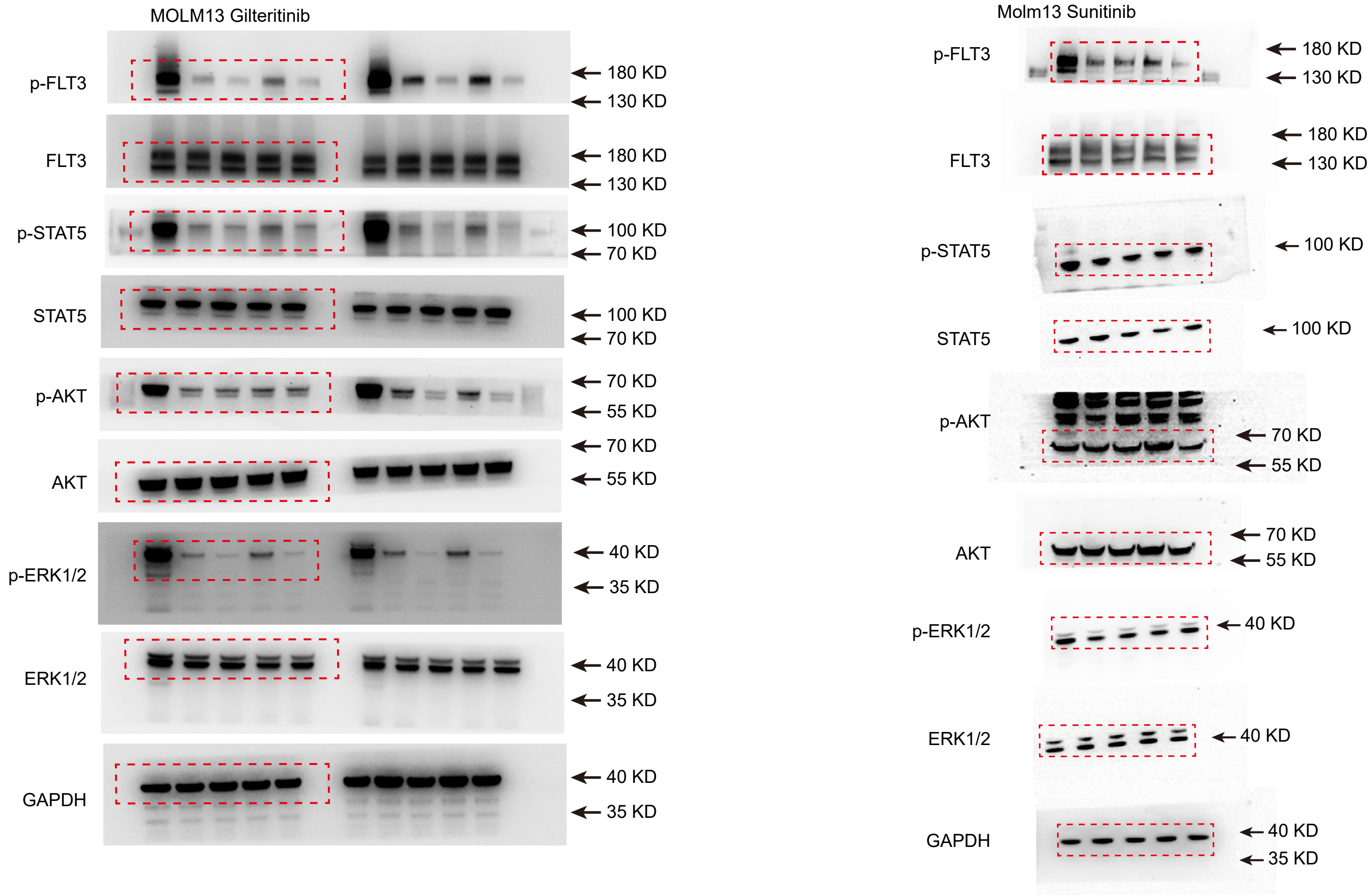

Full unedited blot for Figure 4J

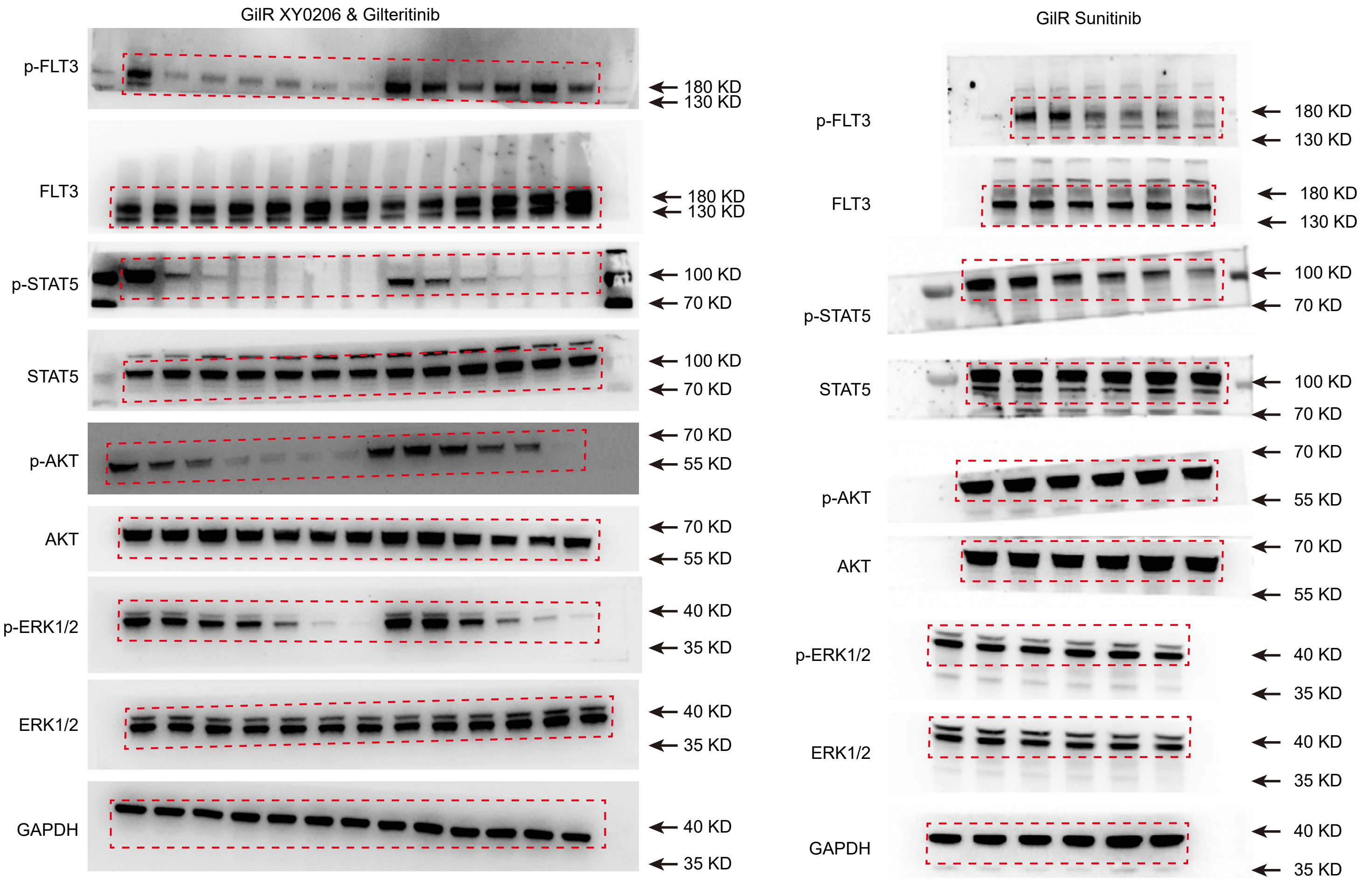

Full unedited blot for Figure 5D

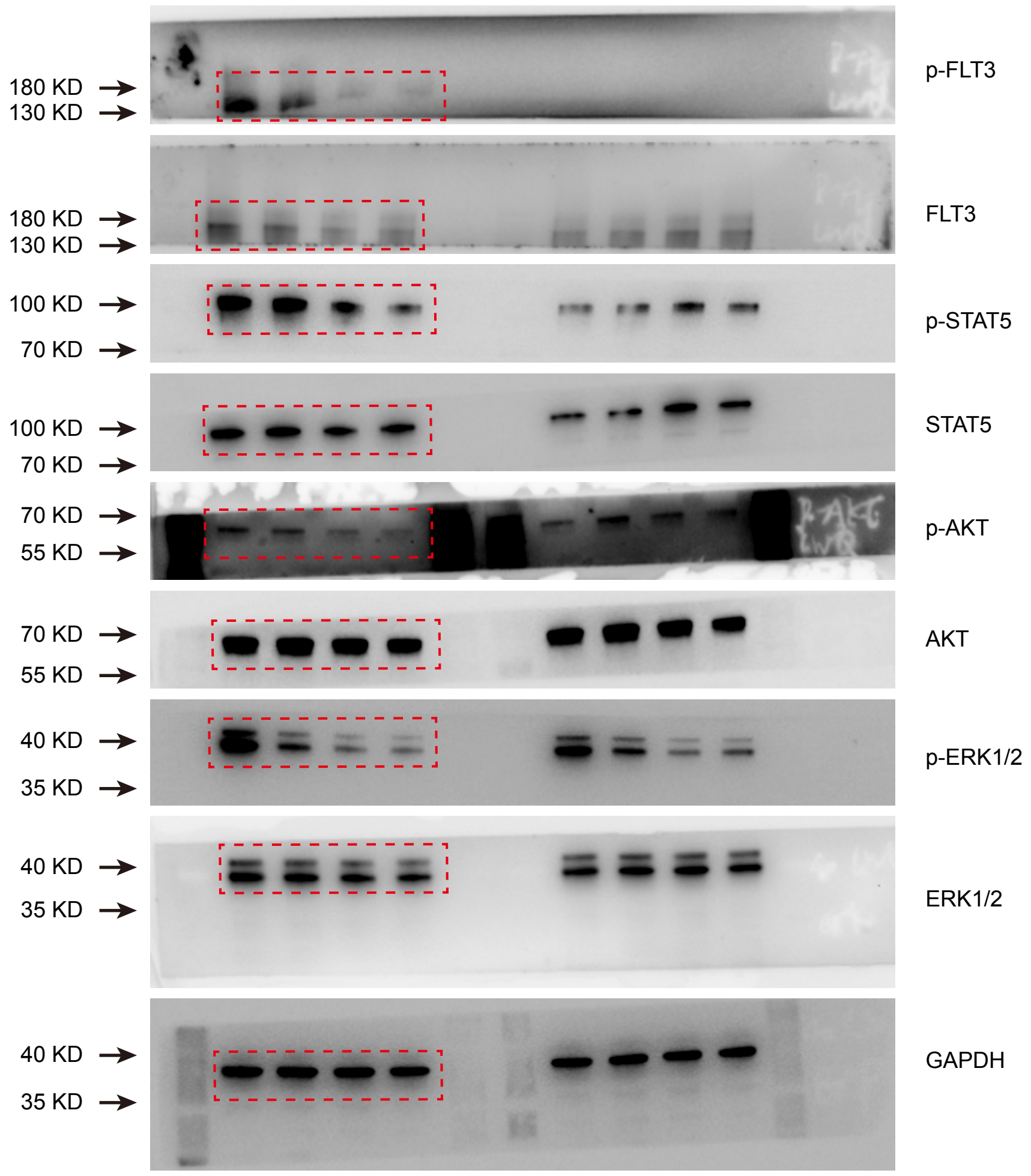

Full unedited blot for Supplemental Figure 2B

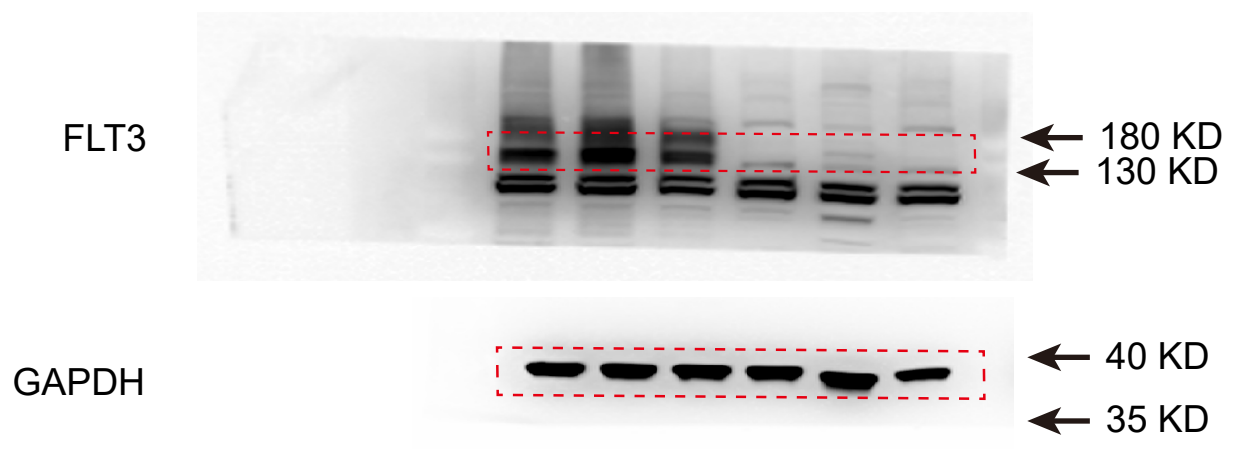

Full unedited blot for Supplemental Figure 2I

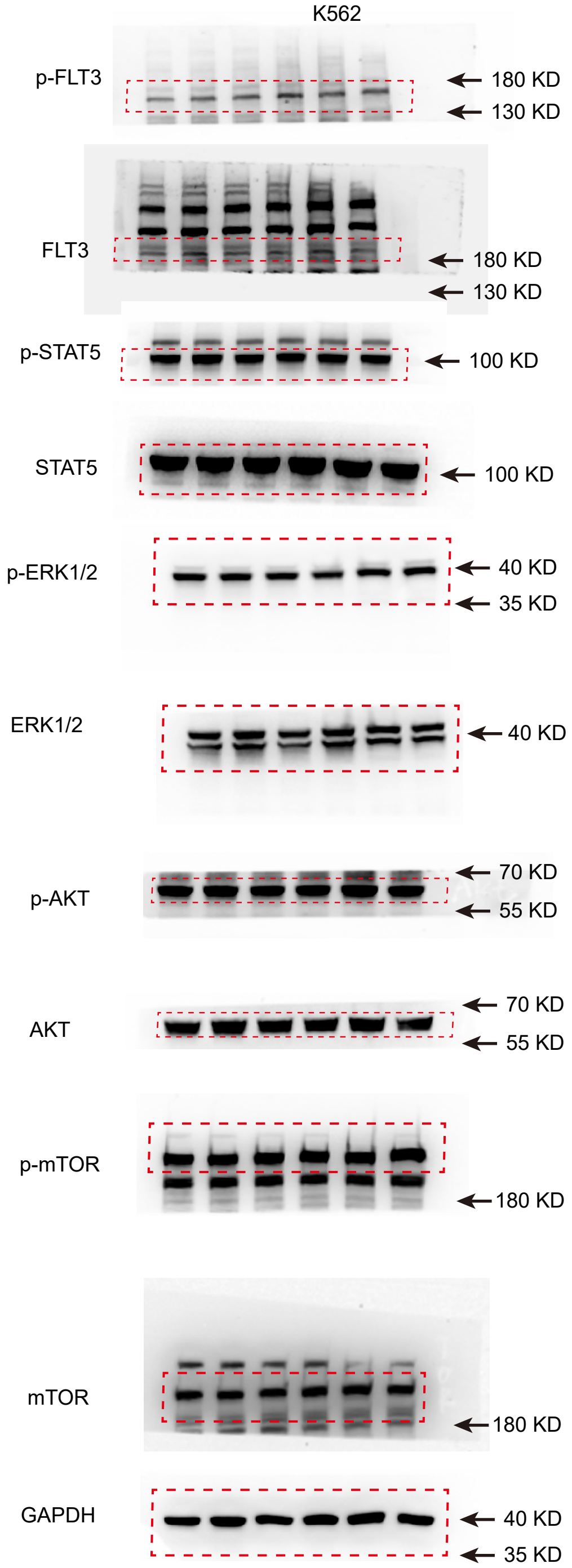

Full unedited blot for Supplemental Figure 3A

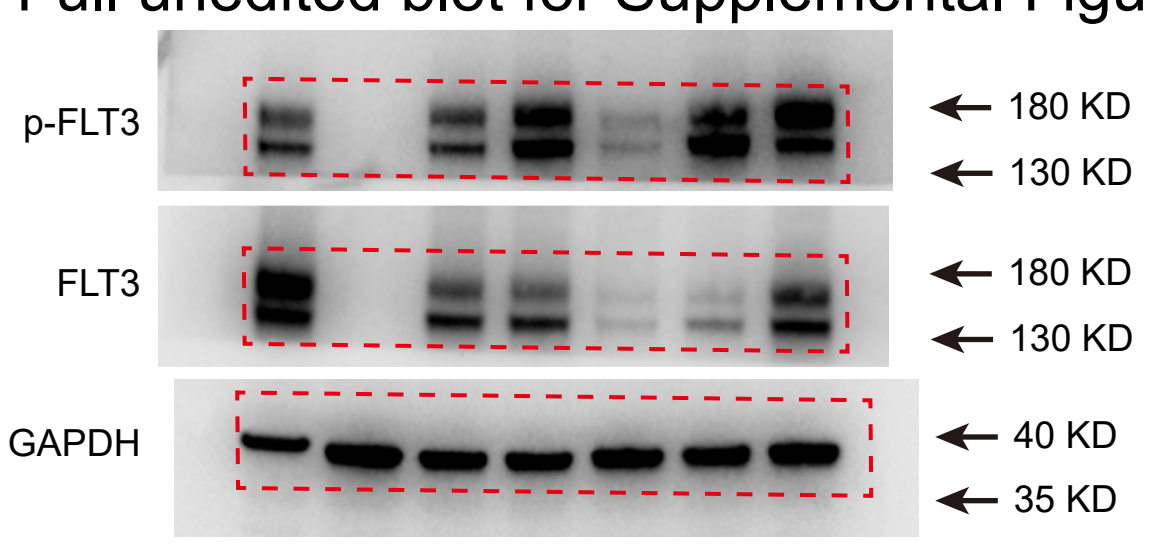

Full unedited blot for Supplemental Figure 4C

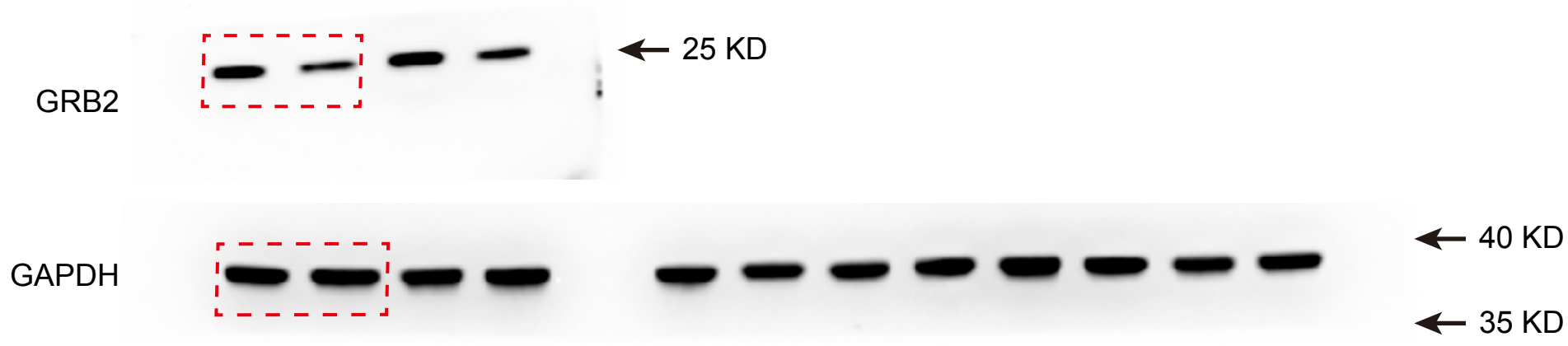

Full unedited blot for Supplemental Figure 4D

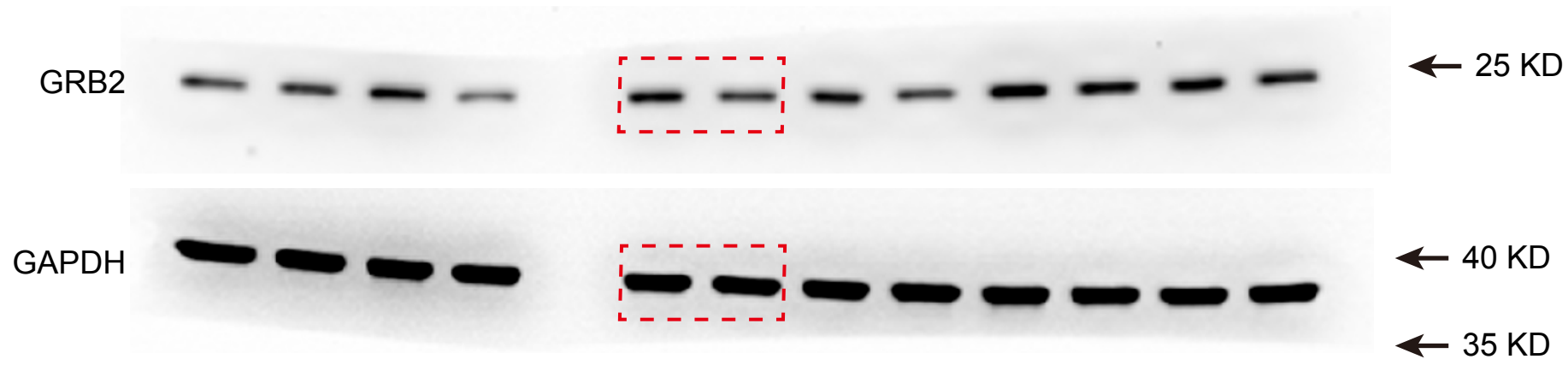

Full unedited blot for Supplemental Figure 4E

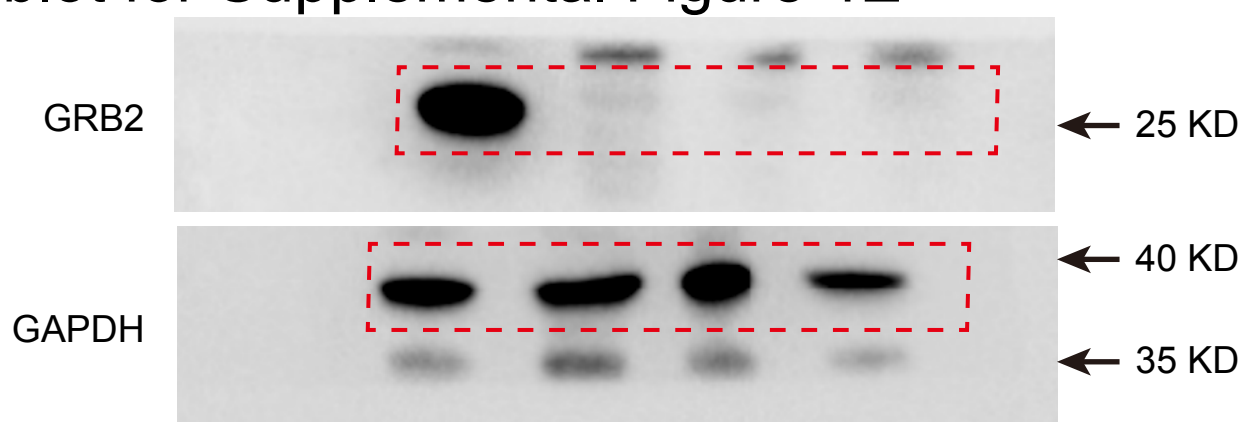

Supplement: Supplementary file 2 [file jci-136-204701-s199.pdf]
